# Haploinsufficiency of *Syngap1* in Striatal Indirect Pathway Neurons Alters Motor and Goal-Directed Behaviors in Mice

**DOI:** 10.1523/JNEUROSCI.1264-23.2024

**Published:** 2024-10-02

**Authors:** Laura M. Haetzel, Jillian Iafrati, Katherine R. Cording, Mahmoud Farhan, Sasan D. Noveir, Gavin Rumbaugh, Helen S. Bateup

**Affiliations:** ^1^Helen Wills Neuroscience Institute, University of California, Berkeley, California 94720; ^2^Departments of Molecular and Cell Biology, University of California, Berkeley, California 94720; ^3^Neuroscience, University of California, Berkeley, California 94720; ^4^Departments of Neuroscience and Molecular Medicine, The Herbert Wertheim UF Scripps Institute for Biomedical Innovation & Technology, Jupiter, Florida 33458; ^5^Skaggs Graduate School of Chemical and Biological Sciences, Jupiter, Florida 33458; ^6^Weill Neurohub Investigator, University of California, Berkeley, California 94720

**Keywords:** autism spectrum disorder, indirect pathway, striatal projection neuron (SPN), striatum, SynGAP, *Syngap1*

## Abstract

*SYNGAP1* is a high-confidence autism spectrum disorder (ASD) risk gene, and mutations in *SYNGAP1* lead to a neurodevelopmental disorder (NDD) that presents with epilepsy, ASD, motor developmental delay, and intellectual disability. *SYNGAP1* codes for Ras/Rap GTP-ase activating protein SynGAP (SynGAP). SynGAP is located in the postsynaptic density of glutamatergic synapses and regulates glutamate receptor trafficking in an activity-dependent manner. In addition to forebrain glutamatergic neurons, *Syngap1* is highly expressed in the striatum, although the functions of SynGAP in the striatum have not been extensively studied. Here we show that *Syngap1* is expressed in both direct and indirect pathway striatal projection neurons (dSPNs and iSPNs) in mice of both sexes. In a mouse model of *Syngap1* haploinsufficiency, dendritic spine density, morphology, and intrinsic excitability are altered primarily in iSPNs, but not dSPNs. At the behavioral level, SynGAP reduction alters striatal-dependent motor learning and goal-directed behavior. Several behavioral phenotypes are reproduced by iSPN-specific *Syngap1* reduction and, in turn, prevented by iSPN-specific *Syngap1* rescue. These results establish the importance of SynGAP to striatal neuron function and pinpoint the indirect pathway as a key circuit in the neurobiology of *SYNGAP1*-related NDD.

## Significance Statement

*SYNGAP1* mutations cause a neurodevelopmental disorder presenting with intellectual disability, motor problems, epilepsy, autism spectrum disorder, and a constellation of other behavioral and psychiatric conditions. SynGAP protein is highly expressed in the striatum, but its functions in this brain region have not yet been explored. This study shows that loss of one copy of the *Syngap1* gene from striatal indirect, but not direct, pathway neurons alters synaptic properties, cellular excitability, motor behaviors, and goal-directed responding in mice. This work provides a new perspective on the functions of SynGAP and suggests that altered activity in striatal circuits may be an important driver of the motor and learning alterations in people with *SYNGAP1* disorder.

## Introduction

Mutations in the *SYNGAP1* gene lead to a neurodevelopmental disorder (NDD) that presents with intellectual disability (ID), epilepsy, global developmental delays, and autism spectrum disorder (ASD; Hamdan et al., 2009; Berryer et al., 2013; Vlaskamp et al., 2019). *SYNGAP1* mutations arise de novo in the germline and are thought to lead to loss of function of one copy of the gene (haploinsufficiency). *SYNGAP1* mutations account for an estimated 1% of total nonsyndromic ID cases (Hamdan et al., 2009; Gamache et al., 2020) and there is currently no targeted treatment for *SYNGAP1*-related NDD.

*SYNGAP1* codes for Ras/Rap GTP-ase activating protein SynGAP (SynGAP), which facilitates the hydrolysis of GDP to GTP, thereby inhibiting the activity of Ras, Rap, and their downstream signaling targets (Chen et al., 1998; Kim et al., 1998; Pena et al., 2008). Under baseline conditions, SynGAP is bound to PSD-95 and serves to inhibit the Ras signaling pathway and the insertion of AMPARs into the postsynaptic membrane (Rumbaugh et al., 2006). In cultured neurons, this “brake” is released during synaptic plasticity, allowing AMPARs to move into the membrane to induce synaptic potentiation (Araki et al., 2015). While SynGAP has been traditionally thought of as a negative regulator of synaptic function, it may potentiate synaptic strength in some contexts due to differing isoform activity or opposing actions of its target molecules, Ras and Rap, on AMPAR trafficking (Zhu et al., 2002; McMahon et al., 2012; Gamache et al., 2020). Recent studies have also indicated roles for SynGAP outside of the synapse in regulating intrinsic membrane properties and early developmental processes (Arora et al., 2022; Birtele et al., 2023).

SynGAP has been primarily studied in forebrain glutamatergic neurons and less is known about its functions in other cell types (Jeyabalan and Clement, 2016; Gamache et al., 2020). In mice, SynGAP is highly expressed in the striatum where its expression increases sixfold in the first few postnatal weeks (Gou et al., 2020). Alterations in striatal circuitry are strongly implicated in ASD-related behaviors, in particular the repetitive, restricted, and inflexible behaviors, due to the striatum's role in action selection and motor learning (Fuccillo, 2016; Li and Pozzo-Miller, 2020). Studying the function of SynGAP in the striatum is therefore important for understanding disease mechanisms of *SYNGAP1*-related NDD.

The striatum is the major input structure of the basal ganglia, which is primarily composed of two types of GABAergic striatal projection neurons (SPNs). SPNs receive glutamatergic inputs from the cortex and thalamus, dopaminergic inputs from the midbrain, and various local inhibitory inputs (Gerfen and Surmeier, 2011). Direct pathway SPNs (dSPNs) predominantly express D1-type dopamine receptors (D1Rs) and send outputs to the substantia nigra pars reticulata (SNr; Gerfen and Surmeier, 2011), which are classically thought to promote locomotor activity and action selection (Bateup et al., 2010; Kravitz et al., 2010; Tai et al., 2012). Indirect pathway SPNs (iSPNs) project to the external globus pallidus and express D2-type dopamine receptors and adenosine 2A (A2A) receptors (Gerfen and Surmeier, 2011). Bulk activation of iSPNs suppresses locomotor activity (Kravitz et al., 2010); however, our understanding of indirect pathway function is still evolving (Isett et al., 2023). Coordinated activity of both striatal pathways is integral to action selection and motor control (Tecuapetla et al., 2016; Cox and Witten, 2019).

The goal of this study was to assess *Syngap1 *expression in dSPNs and iSPNs and investigate how striatal pathway-specific SynGAP reduction affects cellular properties and striatal-dependent behaviors. We found that *Syngap1* mRNA is highly expressed in both types of SPNs and that haploinsufficiency of *Syngap1* in mice alters the synaptic and physiological properties of iSPNs. Consistent with this, reduction of *Syngap1* expression in iSPNs led to altered rotarod motor performance and goal-directed responding. Conversely, selective restoration of *Syngap1* expression in iSPNs prevented behavioral alterations in global *Syngap1* haploinsufficient mice. These findings demonstrate that SynGAP is an important regulator of SPN function and pinpoint iSPNs as potential mediators of behavioral alterations in *SYNGAP1-*related NDD.

## Materials and Methods

### Mice

Animal experiments were performed in accordance with protocols approved by the University of California, Berkeley, Institutional Animal Care and Use Committee (protocol #: AUP-2016-04-8684-2). Both male and female mice were used across all experiments and were postnatal day (P) 40–80 unless otherwise stated. *Syngap1^lx-st^* conditional rescue mice were provided by Dr. Gavin Rumbaugh (Jackson Laboratory strain #029304) and are haploinsufficient for *Syngap1* in the absence of Cre (Clement et al., 2012). *Syngap1^fl^* conditional knock-out mice (Jackson Laboratory strain #029303) have *loxP* sites flanking exons 6–7 of the *Syngap1* gene, leading to excision and loss-of-function in the presence of Cre (Clement et al., 2012). Other lines used in this study were *Drd2-EGFP* (GENSAT [MMRRC #000230-UNC]; Gong et al., 2003), *Drd1-Cre* (EY217; GENSAT [MMRRC #030778-UCD]; Gong et al., 2007), and *Adora2a-Cre* (KG139; GENSAT [MMRRC #031168-UCD]; Gong et al., 2007).

### Fluorescent in situ hybridization

Fluorescent in situ hybridization was performed to quantify *Syngap1* mRNA expression in iSPNs and dSPNs. Brains were harvested, flash-frozen in OCT mounting medium (Fisher Healthcare #23-730-571) on dry ice, and stored at −80°C for up to 6 months. Then, 12–18 µm sections were collected using a cryostat, mounted directly onto 75 × 25 mm Superfrost Plus glass slides (VWR #48311-703) and stored at −80°C for up to 6 months. In situ hybridization was performed according to the protocols provided with the RNAscope Multiplex Fluorescent Reagent Kit (ACD #323100). *Syngap1* mRNA was visualized with a probe in channel 1 (ACD #417381), *Drd2* mRNA in channel 2 (ACD #406491-C2), and *Drd1* mRNA in channel 3 (ACD #406581-C3). After incubations, sections were secured on slides using VectaShield HardSet mounting medium with DAPI (VWR #101098-050) and 60 × 24 mm rectangular glass coverslips (VWR #16004-096). Sections were imaged on an Olympus FluoView 3000 confocal microscope using Olympus UPlanSApo 20×/0.75 (Olympus #1-U2B825) or Olympus UPlanSApo 60×/1.35 oil objectives (Olympus #1-U2B832, for quantification). Images were analyzed using FIJI. A threshold of 2% neuron area was used to determine whether a cell was *Syngap1* positive.

### Western blotting

Mice were deeply anesthetized using isoflurane and decapitated. Brains were dissected on ice, and dorsal striatum punches were collected from both hemispheres. The tissue was flash-frozen in liquid nitrogen and stored at −80°C. On the day of the experiment, frozen samples were sonicated until homogenized (QSonica Q55) in 500 μl lysis buffer containing 1% SDS in 1× PBS with Halt phosphatase inhibitor cocktail (Thermo Scientific #78420) and Complete mini EDTA-free protease inhibitor cocktail (Roche #4693159001). Sample homogenates were then boiled on a heat block at 95°C for 10 min and cooled to room temperature. Total protein content was determined by BCA assay (Thermo Scientific #23227). Following BCA assay, protein homogenates were mixed with 4× Laemmli sample buffer (Bio-Rad #161-0747). Proteins (10–15 μg) were loaded onto 4–15% Criterion TGX gels (Bio-Rad #5671084). Proteins were transferred to PVDF membrane (Bio-Rad #1620177) at 4°C overnight using the Bio-Rad Criterion Blotter (12 V constant voltage). The membranes were blocked in 5% milk in 1× TBS with 1% Tween (TBS-T) for 1 h at room temperature and incubated with primary antibodies against SynGAP (Invitrogen #PA1-046, 1 μg/ml stock diluted to 0.2 μg/ml) or Histone 3 (Cell Signaling #3638, 1:5,000) diluted in 5% milk in TBS-T overnight at 4°C. The following day, after 3 × 10 min washes with TBS-T, the membranes were incubated with horseradish peroxidase-conjugated secondary antibodies (Bio-Rad, #1705047 or #1705046, 1:5,000) for 1 h at room temperature in 5% milk in TSB-T. Following 6 × 10 min washes with TBS-T, the membranes were incubated with chemiluminescence substrate (PerkinElmer #NEL105001EA) for 1 min and exposed to GE Amersham Hyperfilm ECL (VWR #95017-661). Membranes were stripped with ReBlot Plus Strong solution (Millipore #2504) to reblot on subsequent days.

Western blot analysis was performed blind to genotype. Bands were quantified by densitometry using ImageJ (NIH) software. Histone 3 was used as a loading control. Two striatum samples (left and right hemispheres) were analyzed per mouse and averaged together. Once quantified, protein content was expressed as percentage of WT within a given experiment.

### Spine morphology reconstruction

Fixed tissue microinjections were performed to assess dendritic spine morphology in iSPNs and dSPNs (Dumitriu et al., 2011). Young adult *Syngap1^+/lx-st^* mice expressing *Drd2-EGFP* were transcardially perfused with 1% paraformaldehyde in 0.1 PB, followed by 4% paraformaldehyde in 0.1 PB. Brains were harvested and postfixed overnight in 4% paraformaldehyde in 0.1 PB and then stored in 0.1 PB with 0.1% w/v sodium azide for up to 3 months. Then, 250 µm coronal sections were collected using a vibratome and stored in 0.1 PB with 0.1% w/v sodium azide for less than a week before microinjection and mounting. Striatal sections were visualized on a Scientifica SliceScope in 1× PBS with a 60× water immersion objective (Olympus #LUMPFLN60XW) to identify D2-GFP-positive cells (iSPNs) or D2-GFP-negative cells (putative dSPNs, cells with aspiny dendrites were considered interneurons and discarded). Cells were injected with a sharp glass electrode (100–150 MΩ) filled with 100 mM Lucifer yellow in 200 mM KCl (Molecular Probes #L453). Once dye began to diffuse into the cell, −2 nA of current was applied for 15 min using an Axon Instruments MultiClamp 700B amplifier. 7–10 SPNs in the dorsolateral striatum were injected before the section was mounted onto a glass slide using VectaShield HardSet mounting medium without DAPI (Vector Labs #H-1400-10).

Dendrites were imaged on an Olympus FluoView 3000 scanning confocal microscope at 60× magnification with 2.5× digital zoom for a final magnification of 150×. Images were deconvoluted in the CellSens4 software using a built-in advanced maximum likelihood algorithm. Dendritic spine reconstructions were generated using the filament tracer feature in Imaris 9.3.1. One to three dendrites were reconstructed per cell, with the detection parameters for thinnest spine diameter set to 1.5 µm and the maximum spine length set to 3.5 µm. After automatic detection of spines, the digital reconstruction was compared with the original *z*-stack image to ensure accuracy. Detected spines were manually reviewed by the experimenter and added or removed as needed. Spine density (spines/µm), spine head diameter (µm), spine area (µm^2^), spine length (µm), and spine neck length (µm) values were extracted from the exported Imaris reconstruction statistics files using custom Python code (https://github.com/lhaetzel/imaris_extraction_code).

### Behavioral experiments

Mice in behavior experiments were housed on a reverse light/dark cycle. Behavior studies were carried out in the dark phase of the light cycle under dimmed white lights. Mice were habituated to the behavior testing room for at least 30 min prior to testing and at least 24 h elapsed between sessions. All behavior equipment was cleaned between each trial with 70% ethanol. Equipment was rinsed with diluted soap and water at the end of each day. Additionally, male mice were trained or tested before female mice each day. Experimenters were blinded to genotype during behavioral testing.

#### Open field

Exploratory behavior in a novel environment and general locomotor activity were assessed by a 10 min session in an open-field chamber (40 cm L × 40 cm W × 34 cm H) made of transparent plexiglass. Horizontal infrared photobeams were positioned at 5 cm above the floor to detect rearing. The mouse was placed in the bottom right-hand corner of the arena and behavior was recorded using an overhead camera and analyzed using the ANY-maze (Stoelting) behavior tracking software. A central square (40% of the open-field area) was defined in ANY-maze to quantify the time spent in the center of the arena.

#### Rotarod

The accelerating rotarod test was used to examine motor coordination and motor learning. On the first testing day, mice were habituated to the rotarod apparatus (Ugo Basile #47650) for 1 min at minimal constant speed and then trained over 4 consecutive days. On each training day, animals completed three, 5 min trials with a 5–10 min break between trials. The rotarod was accelerated from 5 to 80 revolutions per minute (rpm) over 300 s for all trials (12 trials total, spread across 4 testing days). Terminal speed for each trial was defined as the maximal rotating speed an animal reached before falling off the rotarod. Initial performance was defined as the terminal speed on trial 1. Learning rate was calculated as the slope of performance (measured as terminal speed) from trial 1 to trial 12 for each individual mouse. For mice undergoing rotarod testing, the home cages were not supplemented with running wheels.

#### Operant conditioning

Goal-directed behavior was assessed in an operant conditioning lever pressing task using a random ratio (RR) reinforcement schedule with a food pellet reinforcer followed by outcome revaluation testing (Gremel and Costa, 2013). Animals were food restricted to ∼95% of their body weight before commencing training in operant conditioning chambers (Med Associates #ENV-307A). The chambers contained a retractable lever on either side of the food receptacle and a house light on the opposite end of the chamber. Each session began with illumination of the house light and the presentation of the left lever. The session ended with retraction of the lever and with the house light turning off. The lever remained present throughout the session. Mice were weighed before each session and habituated to the experiment room for 30 min.

During the first training session, a food pellet (20 mg regular “chow” pellet, Bio-Serve #F05684) was delivered every 60 s, on average, for a total of 15 min with no levers present. During the continuous reinforcement (CRF) sessions, mice were presented with the left lever. Each lever press was rewarded for a maximum of 5 (CRF5, 1 session), then 15 (CRF15, 1 session), and finally 30 (CRF30) rewards per each session. The session concluded once the mouse obtained the maximum number of reinforcers or once 60 min elapsed. Once the mice earned at least seven pellets in CRF30, they moved on to the RR sessions the next day. Mice that failed to earn at least seven reinforcers after two sessions of CRF30 were excluded from further testing.

During RR training, mice began with RR10 (1 in 10 chance of pellet delivery for every lever press) for two sessions and then moved to RR20 (1 in 20 chance of pellet delivery for every lever press) for four sessions. Lever presses per minute were quantified during each session from CRF5 to RR20. For all RR sessions, the actual reward probability for each mouse was calculated and compared with the expected reward probability (i.e., 1 in 10 or 1 in 20). The deviation from expected probability was summed across the six RR training days and mice that had a total deviation of >45 were excluded from the analysis.

Outcome devaluation testing began the day after the last RR20 session. On the devalued day, mice were given 1 h *ad libitum* access to food pellets prior to being placed in the operant conditioning chamber for 5 min with no reinforcer delivered in response to lever pressing. On the valued day, mice were given 1 h *ad libitum* access to a different reinforcer (20% sucrose in water) and underwent the same nonreinforced probe test as on the devalued day.

The devaluation index ([lever presses valued state − lever presses devalued state] / [lever presses valued state + lever presses devalued state]) was computed as a measure of goal-directed behavior.

#### Three-chamber social approach test

Social approach behavior was assessed using the three-chamber social approach test. The testing apparatus consisted of clear Plexiglas (25 cm L × 58 cm W × 26 cm H) with walls dividing the apparatus into to three chambers. Inverted wire cups (8 cm diameter and 11 cm tall) were placed into the left and right chambers. The test mouse was placed in the center chamber and allowed to explore and habituate to all three chambers for 10 min. The mouse was then briefly placed in a holding cage. During this time, a novel sex-matched juvenile (3–5 week old) C57BL/6J mouse was placed under a wire cup in one of the side chambers. The test mouse was then placed in the center chamber and allowed to explore the three chambers for 10 min. The location of the novel mouse and the empty wire cup was alternated between mice. Behavior was recorded using a video camera positioned directly above the center of the apparatus and analyzed using the ANY-maze (Stoelting) behavior tracking software. The apparatus and wire cups were cleaned with 70% ethanol between mice.

### Electrophysiology

For the intrinsic excitability experiments in Figure 3, mice (P40–60) were transcardially perfused for ∼10 s with ice-cold NMDG-based recovery solution, pH 7.4 (adjusted with 10 M HCl; in mM): 93 NMDG, 2.5 KCl, 1.2, NaH_2_PO_4_, 30 NaHCO_3_, 20 HEPES, 25 dextrose monohydrate, 5 L-ascorbic acid, 3 myo-inositol, 3 Na-pyruvate, 10 MgCl_2_, and 0.5 CaCl_2_ (Ting et al., 2018). Following perfusion, the brain was rapidly removed, and 275 µm coronal sections were cut using a vibratome (Leica #VT100S) in the NMDG-based recovery solution. Slices were incubated in NMDG solution for 9–11 min, depending on the age of the animal, at 34°C, and then transferred to a second holding chamber with room temperature ACSF. NMDG-based recovery solution and ACSF were bubbled continuously with carbogen (95% O_2_ and 5% CO_2_) and osmolarity was kept between 300 and 310 mOsm.

For the miniature excitatory postsynaptic current recordings (mEPSCs, Fig. 3), intrinsic excitability experiments in Figure 4, and rescue intrinsic excitability experiments in Figure 10, mice (P40–60) were perfused for ∼30 s with ice-cold artificial cerebrospinal fluid (ACSF), pH 7.4, containing the following (in mM): 127 NaCl, 25 NaHCO_3_, 1.25 NaH_2_PO_4_, 2.5 KCl, 1 MgCl_2_, 2 CaCl_2_, and 25 glucose. Following perfusion, the brain was rapidly removed, and 275 µm coronal sections were cut using a vibratome (Leica #VT100S) in a choline-based external solution, pH 7.8, containing the following (in mM): 110 choline chloride, 25 NaHCO_3_, 1.25 NaHPO_4_, 2.5 KCl, 7 MgCl_2_, 0.5 CaCl_2_, 25 glucose, 11.6 sodium ascorbate, and 3.1 sodium pyruvate. Slices were incubated in ACSF for 15 min at 36°C and then allowed to return to room temperature for at least 15 min before recording.

All recordings were made with a MultiClamp 700B amplifier (Molecular Devices) in a recording chamber (Scientifica) with room temperature oxygenated ACSF, and slices were secured with a slice anchor. The resistance of glass patch electrodes (Sutter #BF150-86-7.5) was kept between 3 and 5 ohms.

#### Current-clamp recordings

Current-clamp recordings were made using a potassium-based internal solution, pH 7.4, containing the following (in mM): 135 KMeSO_4_, 5 KCl, 5 HEPES, 4 Mg-ATP, 0.3 Na-GTP, 10 phosphocreatine, and 1 EGTA. To block synaptic transmission, NBQX (10 µM, Tocris #1044), CPP (10 µM, Tocris #0247), and picrotoxin (50 µM, Abcam #ab120315) were added to the external solution. One-second-long depolarizing current steps were applied to elicit action potentials. For intrinsic excitability experiments in Figure 3, rheobase was found using 10 pA current steps. The sampling rate was then increased to 50 kHz to acquire a spike train for action potential shape analysis at 10 pA above rheobase. Subsequently, the sampling rate was reduced to 10 kHz and incremental 50 pA current steps were applied to generate the input/output curve.

For intrinsic excitability experiments in Figures 4 and 10, the first current step that evoked at least one action potential was considered the rheobase current, and sampling rate was not changed when considering action potentials for shape analysis. A cell was classified as being in depolarization block at a given current step if the number of action potentials fired at that current step was at least two fewer than the number fired at the previous current step. For all current-clamp experiments, no holding current was applied to the membrane, and cells with a resting membrane potential above −65 mV were excluded.

#### Voltage-clamp recordings

Voltage-clamp recordings were made using a cesium-based internal solution, pH 7.4, containing the following (in mM): 120 CsMeSO_4_, 15 CsCl, 10 TEA-Cl, 8 NaCl, 10 HEPES, 1 EGTA, 5 QX-314, 4 Mg-ATP, and 0.3 Na-GTP. mEPSCs were recorded in the presence of picrotoxin (50 μM, Abcam #ab120315) and tetrodotoxin (1 μM, Abcam #ab120055) to isolate excitatory synaptic events and prevent action potential-mediated release. Recordings were acquired with the amplifier Bessel filter set at 3 kHz.

Data were acquired using ScanImage software (https://github.com/bernardosabatini/SabalabAcq) and analyzed in Igor Pro (WaveMetrics). Recordings with a series resistance >25 MΩ were excluded.

### Statistical analysis

GraphPad Prism versions 9 and 10 were used to perform statistical analyses. Two-tailed unpaired *t* tests were used for comparisons between two groups. For data that did not pass the D’Agostino and Pearson’s normality test, a two-tailed Mann–Whitney test was used. A one-way ANOVA with Holm–Sidak's post hoc test was used to compare the means of three or more groups. For data that did not pass the D’Agostino and Pearson’s normality test, a Kruskal–Wallis test with Dunn's post hoc tests was used. Repeated-measures (RM) two-way ANOVAs or mixed-effects analyses were used to compare differences between groups for experiments with two independent variables. A Wilcoxon matched-pairs signed rank test was used to analyze paired samples. *p* values were corrected for multiple comparisons. Statistical significance was defined as follows: *p < 0.05, **p < 0.01, ***p < 0.001, ****p < 0.0001.

## Results

### *Syngap1* mRNA and SynGAP protein are expressed in SPNs of adult mice

**Figure 1.**
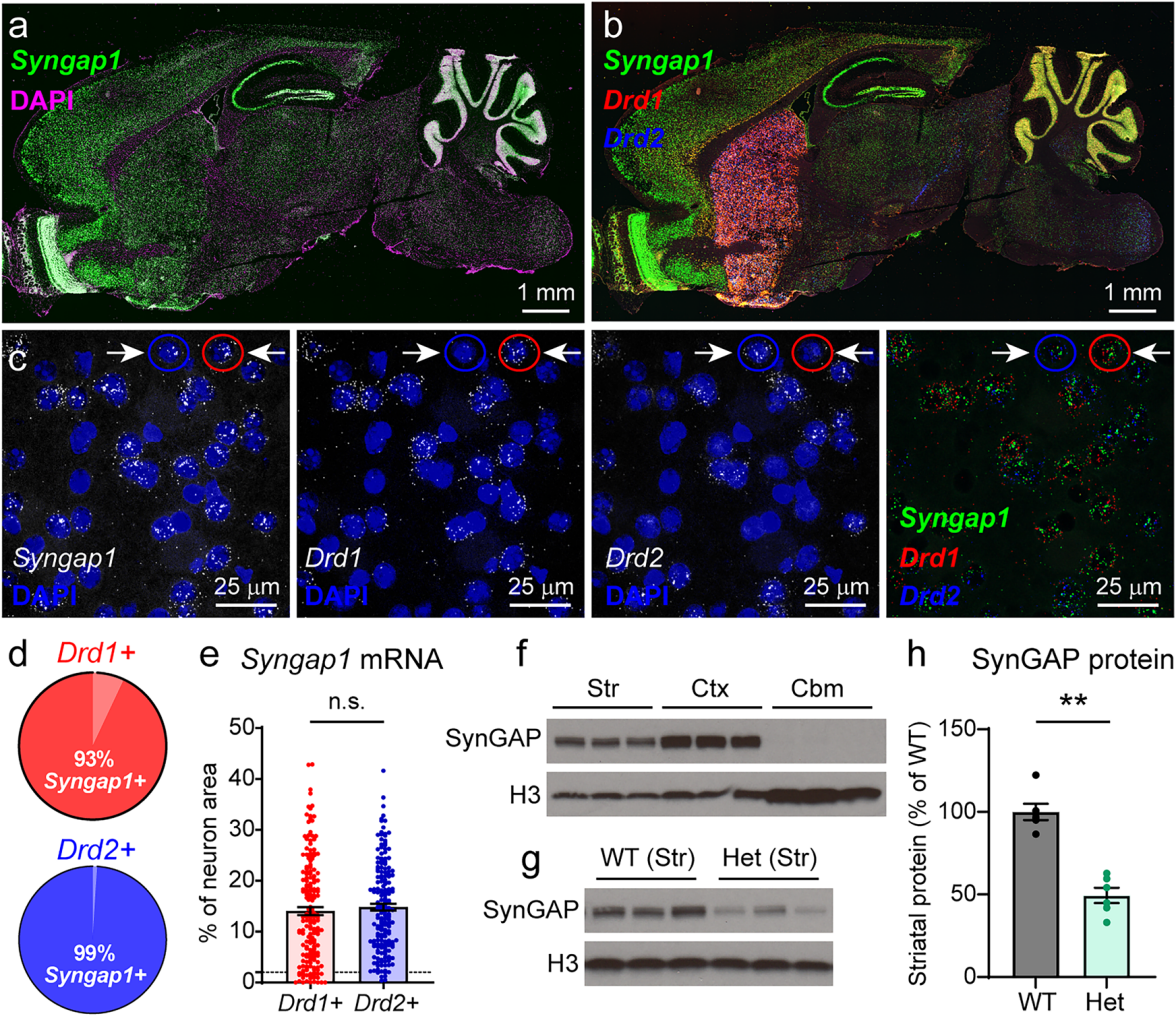
*Syngap1* mRNA and SynGAP protein are expressed in the striatum of adult mice. ***a***, Representative sagittal brain section from an adult wild-type mouse. In situ hybridization for *Syngap1* is in green, and DAPI is in magenta. Scale bar, 1 mm. ***b***, Same section as in ***a*** with *Syngap1* in green, *Drd1* in red, and *Drd2* in blue. ***c***, Zoomed-in images of cells in the dorsolateral striatum showing *Syngap1*, *Drd1*, and *Drd2* mRNA together with the nuclear stain DAPI. The right panel shows a merged fluorescence image without DAPI. Scale bars, 25 µm. The red circle shows an example *Drd1+/Syngap1+* cell, and the blue circle shows a *Drd2+/Syngap1+* cell. ***d***, Proportion of *Drd1+* and *Drd2+* cells expressing *Syngap1* mRNA. ***e***, Quantification of *Syngap1* mRNA expressed as the percent of neuron area in *Drd1+* and *Drd2+* striatal cells (mean ± SEM, dots represent values for individual cells). Mann–Whitney test, *p *= 0.2057, *U *= 14,950, *n *= 180 cells from 3 mice per group. ***f***, Representative Western blot (WB) of lysates from the striatum (Str), cortex (Ctx), and cerebellum (Cbm) of wild-type mice (samples from 3 mice are shown). This experiment was repeated two times. ***g***, Representative WB of striatal lysates from *Syngap1^+/+^* (WT) and *Syngap1^+/lx-st^* (Het) mice (samples from 3 mice per genotype are shown). This experiment was repeated two times. ***h***, Quantification of striatal SynGAP protein levels from *Syngap1* WT and Het mice (mean ± SEM, dots represent values from individual mice). Mann–Whitney test, **p = 0.0022, *U *= 0, *n *= 6 mice per genotype.

In addition to the cortex and hippocampus, SynGAP has been shown to be expressed in the striatum using Western blotting of bulk tissue (Araki et al., 2020; Gou et al., 2020). To determine which striatal neurons express *Syngap1*, we performed fluorescent in situ hybridization for *Syngap1* mRNA and markers of SPN subtypes in the dorsolateral striatum (DLS; Fig. 1*a*,*b*). *Syngap1* mRNA was strongly expressed to a similar extent in both SPN subtypes: direct pathway neurons labeled with *Drd1* mRNA (dSPNs, D1+) and indirect pathway neurons identified by *Drd2* mRNA (iSPNs, D2+). Ninety-three percent of dSPNs and 99% of iSPNs expressed *Syngap1* mRNA (Fig. 1*c*,*d*). There was no difference in the level of *Syngap1* mRNA expressed in dSPNs versus iSPNs (Fig. 1*e*). At the protein level, Western blots of brain lysates from WT mice showed that SynGAP protein is highly expressed in the striatum and cortex, with little to no expression in the cerebellum (Fig. 1*f*), confirming previous results (Araki et al., 2020; Gou et al., 2020).

In humans, *SYNGAP1*-related nonsyndromic intellectual disability (*SYNGAP1*-related NDD) results from heterozygous mutations in *SYNGAP1* (Vlaskamp et al., 2019). To model this in mice, we used a mouse line with a loxP-flanked stop cassette inserted upstream of one copy of the *Syngap1* gene (*Syngap1^+/lx-st^*; Clement et al., 2012). As expected, SynGAP protein was reduced by ∼50% in striatal samples harvested from *Syngap1^+/lx-st^* (Het) mice, relative to their WT littermates (Fig. 1*g*,*h*).

### SynGAP reduction alters dendritic spines in indirect pathway cells

**Figure 2.**
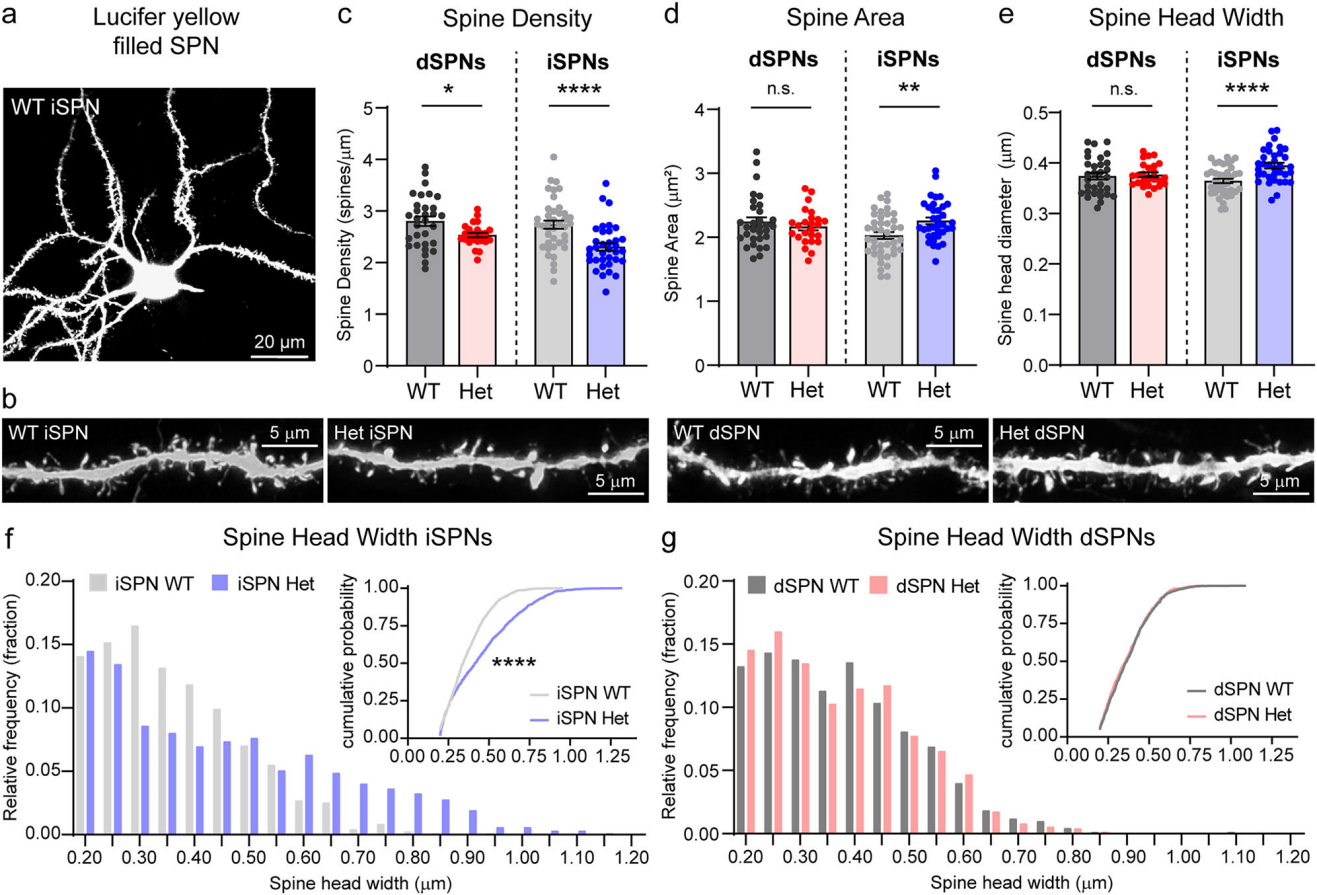
SynGAP reduction alters dendritic spine density and morphology in a cell type-specific manner. ***a***, Example image of a Lucifer yellow dye injected WT iSPN in the dorsolateral striatum. ***b***, Representative images of dendritic spines from WT (*Syngap1^+/+^;D2-GFP+*) and Het (*Syngap1^+/lx-st^*;*D2-GFP*+) iSPNs (left) and WT (*Syngap1^+/+^;D2-GFP-*) and Het (*Syngap1^+/lx-st^*;*D2-GFP*−) dSPNs (right). Scale bars, 5 µm. ***c***, Quantification of spine density in *Syngap1* WT and Het dSPNs and iSPNs (mean ± SEM, dots represent values for individual neurons). dSPN WT versus Het: Welch's two-tailed *t* test, *p = 0.0105, *t *= 2.678, df = 42.60, WT *n *= 31 neurons from 3 mice, Het *n *= 25 neurons from 3 mice. iSPN WT versus Het: Mann–Whitney test, ****p < 0.0001, *U *= 328, WT *n = *40 neurons from 3 mice, Het *n = *35 neurons from 3 mice. ***d***, Mean ± SEM total spine area in *Syngap1* WT and Het dSPNs and iSPNs. dSPN WT versus Het: Mann–Whitney test, *p *= 0.7811, *U *= 370, *n* is the same as for panel ***c***. iSPN WT versus Het: Unpaired two-tailed *t* test, **p = 0.0033, *t *= 3.040, df = 73, *n* is the same as for panel ***c***. ***e***, Mean ± SEM spine head diameter in *Syngap1* WT and Het dSPNs and iSPNs. dSPN WT versus Het: Welch's two-tailed *t* test, *p *= 0.7289, *t *= 0.3485, df = 51.09, *n* is the same as for panel ***c***. iSPN WT versus Het: Unpaired two-tailed *t* test, ****p < 0.0001, *t *= 4.311, df = 73, *n* is the same as for panel ***c***. ***f***, Histogram of spine head width for WT and Het iSPNs. Thirty spines per neuron were randomly selected for this analysis. WT iSPNs (*n *= 1,200 spines from 40 neurons), Het iSPNs (*n *= 1,050 spines from 35 neurons). Inset shows cumulative probability plot of spine head width (same data as in the histogram). Kolmogorov–Smirnov test, ****p < 0.0001, *D *= 0.2229. ***g***, Histogram of spine head width for WT and Het dSPNs. Thirty spines per neuron were randomly selected for this analysis. WT dSPNs (*n *= 930 spines from 31 neurons), Het dSPNs (*n *= 750 spines from 25 neurons). Inset shows cumulative probability plot of spine head width (same data as in the histogram). Kolmogorov–Smirnov test, *p *= 0.3760, *D *= 0.04477.

Since SynGAP is known to be a regulator of glutamatergic synapses, we first investigated how SynGAP reduction affects dendritic spine number and morphology in SPNs. Previous studies have shown that *Syngap1* haploinsufficiency leads to alterations in pyramidal neuron spine morphology and density (Clement et al., 2012; Aceti et al., 2015). To examine this in striatal neurons, we bred *Syngap1^+/lx-st^* mice with *Drd2-EGFP* mice (Gong et al., 2003) to identify iSPNs. We microinjected *Drd2-EGFP-*positive (iSPNs) and *Drd2-EGFP*-negative (putative dSPNs) cells in slices from the DLS with Lucifer yellow dye and imaged dendritic spines using high-resolution confocal microscopy (Fig. 2*a*,*b*). We chose to focus on the DLS as this is the striatal region that is important for motor and habit learning (H. H. Yin et al., 2004, 2009; Gremel and Costa, 2013). We hypothesized that changes in the properties of SPNs in the DLS could drive the formation of repetitive and inflexible motor behaviors in *Syngap1^+/−^* mice.

We found that the density of spines on Het SPNs was significantly reduced, with a more pronounced effect in Het-iSPNs (Fig. 2*c*). In terms of spine morphology, we found that overall spine area (Fig. 2*d*) and spine length (spine length: WT iSPN 1.447 ± 0.019 vs Het iSPN 1.541 ± 0.023; unpaired two-tailed *t* test, ***p = 0.0023, *t *= 3.163, df = 73) were significantly increased in iSPN-Het neurons, but not dSPN Hets (Fig. 2*d*; spine length: WT dSPN 1.202 ± 0.028 vs Het dSPN 1.272 ± 0.024; unpaired two-tailed *t* test, *p = *0.8001, *t *= 0.2545, df = 54). This increased spine size in iSPN-Hets was likely driven by increased spine head size (Fig. 2*e*) as spine neck length was not significantly different between WT and Het iSPNs (WT iSPN 0.6240 ± 0.009 vs Het iSPN 0.5269 ± 0.011; unpaired two-tailed *t* test, *p *= 0.0632, *t *= 1.886, df = 73). In line with this, there was an increased proportion of spines with a large diameter head in iSPN-Het neurons compared with WT (Fig. 2*f*). dSPN-Het neurons did not show a significant difference in spine head width (Fig. 2*e*,*g*). Together, these results show that SynGAP reduction alters SPN spine morphology and density in a cell type-specific manner, with Het iSPNs exhibiting increased spine head size but reduced spine density.

### Loss of *Syngap1* affects the synaptic and intrinsic physiology of iSPNs

**Figure 3.**
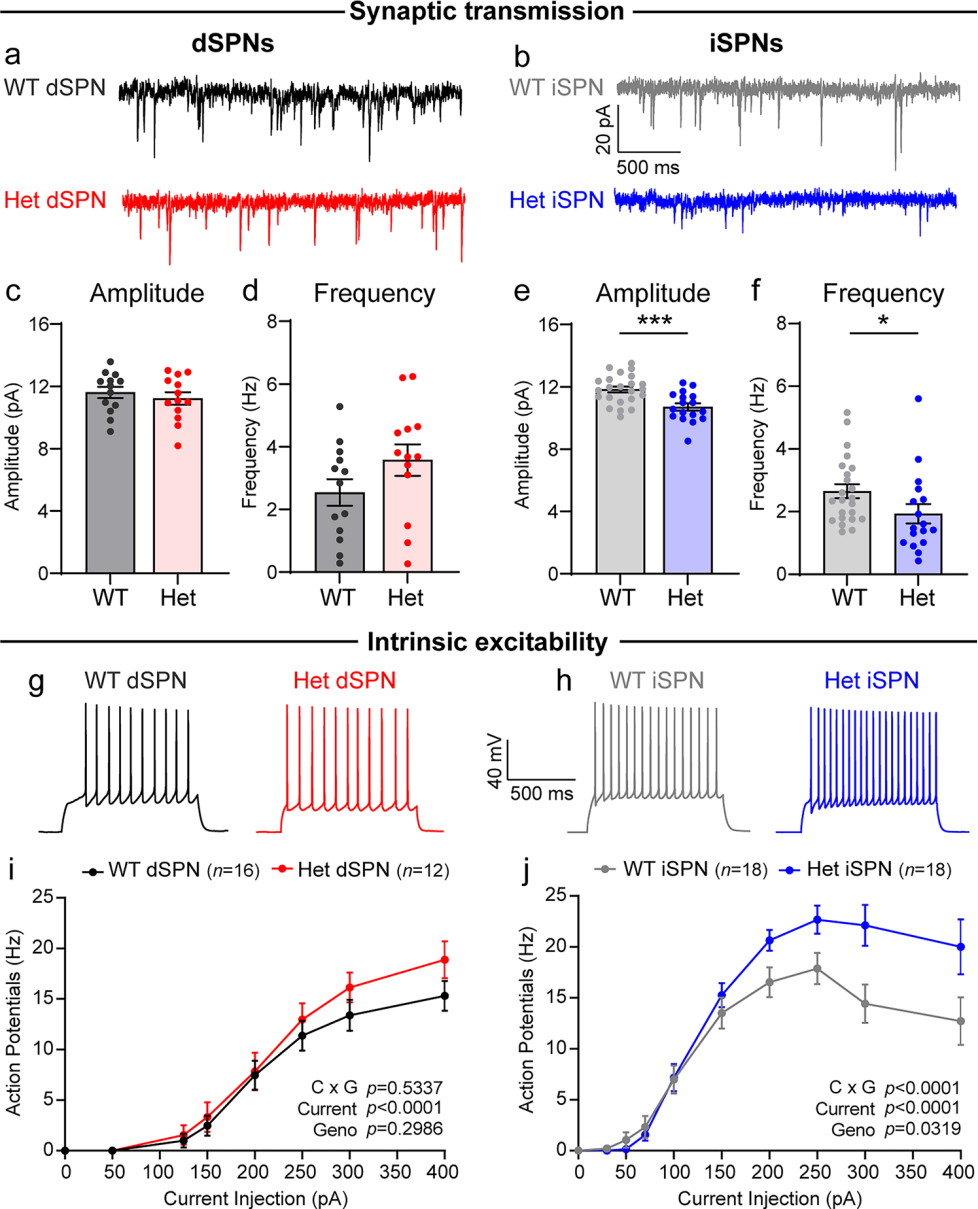
*Syngap1* haploinsufficiency alters synaptic and intrinsic physiology in iSPNs. ***a***, ***b***, Example mEPSC traces recorded from dSPNs (***a***) and iSPNs (***b***) of the indicated genotypes. ***c–f***, Quantification (mean ± SEM) of mEPSC properties in dSPNs (***c***, ***d***) and iSPNs (***e***, ***f***). Dots represent values for individual neurons. ***c***, dSPN mEPSC amplitude: Unpaired two-tailed *t* test, *p *= 0.4777, *t *= 0.7213, df = 24, WT dSPN *n *= 13 neurons from 8 mice, Het dSPN *n *= 13 neurons from 6 mice. ***d***, dSPN mEPSC frequency: Unpaired two-tailed *t* test, *p *= 0.1290, *t *= 1.572, df = 24, *n* is the same as for panel ***c***. ***e***, iSPN mEPSC amplitude: Unpaired two-tailed *t* test, ***p = 0.0007, *t *= 3.700, df = 38, WT iSPN *n *= 23 neurons from 7 mice, Het iSPN *n = *17 neurons from 7 mice. ***f***, iSPN mEPSC frequency: two-tailed Mann–Whitney test, *p = 0.0161, *U *= 108, *n* is the same as panel ***e***. ***g***, ***h***, Sample recordings of action potentials from WT and Het dSPNs (***g***) and iSPNs (***h***) in response to a 200 pA current injection. ***i***, Input–output curve of current injection versus evoked action potentials for *Syngap1* WT (*n *= 16 neurons from 4 mice) and Het (*n *= 12 neurons from 6 mice) dSPNs (mean ± SEM). Two-way repeated-measures ANOVA: current × genotype *p *= 0.5337, *F*_(7,182)_ = 0.8670; current *p *< 0.0001, *F*_(7,182)_ = 95.29; genotype *p *= 0.2986, *F*_(1,26)_ = 1.125; subject *p *< 0.0001 *F*_(26,182)_ = 5.142. ***j***, Input–output curve for *Syngap1* WT (*n *= 18 neurons from 4 mice) and Het (*n* = 18 neurons from 6 mice) iSPNs (mean ± SEM). Two-way repeated-measures ANOVA: current × genotype *p *< 0.0001, *F*_(9,306)_ = 3.926; current *p *< 0.0001, *F*_(2.307,78.44)_ = 96.93; genotype *p *= 0.0319, *F*_(1,34)_ = 5.005; subject *p *< 0.0001, *F*_(34,306)_ = 3.274. See also Extended Data Table 3-1.

To determine how haploinsufficiency of *Syngap1* affects basal synaptic properties, we recorded miniature excitatory postsynaptic currents (mEPSCs) from dSPNs and iSPNs in the DLS of *Syngap1^+/lx-st^;Drd2-EGFP* mice (Fig. 3*a*,*b*). Consistent with the lack of changes in spine density or size, we found no significant differences in the amplitude or frequency of mEPSCs in Het dSPNs compared with WT (Fig. 3*c*,*d*). In contrast, we found a significant reduction in both the average amplitude and frequency of mEPSCs in Het iSPNs (Fig. 3*e*,*f*). Reduced frequency is consistent with the reduction in spine density (Fig. 2*e*) and suggests less glutamatergic inputs onto iSPNs with haploinsufficiency of *Syngap1*. However, the reduction in amplitude is opposite of what would be expected from increased spine head size, which is typically associated with increased postsynaptic strength (Matsuzaki et al., 2001). This suggests that reduction of SynGAP causes a decoupling of spine size and synaptic strength, which could reflect a homeostatic response.

**Figure 4.**
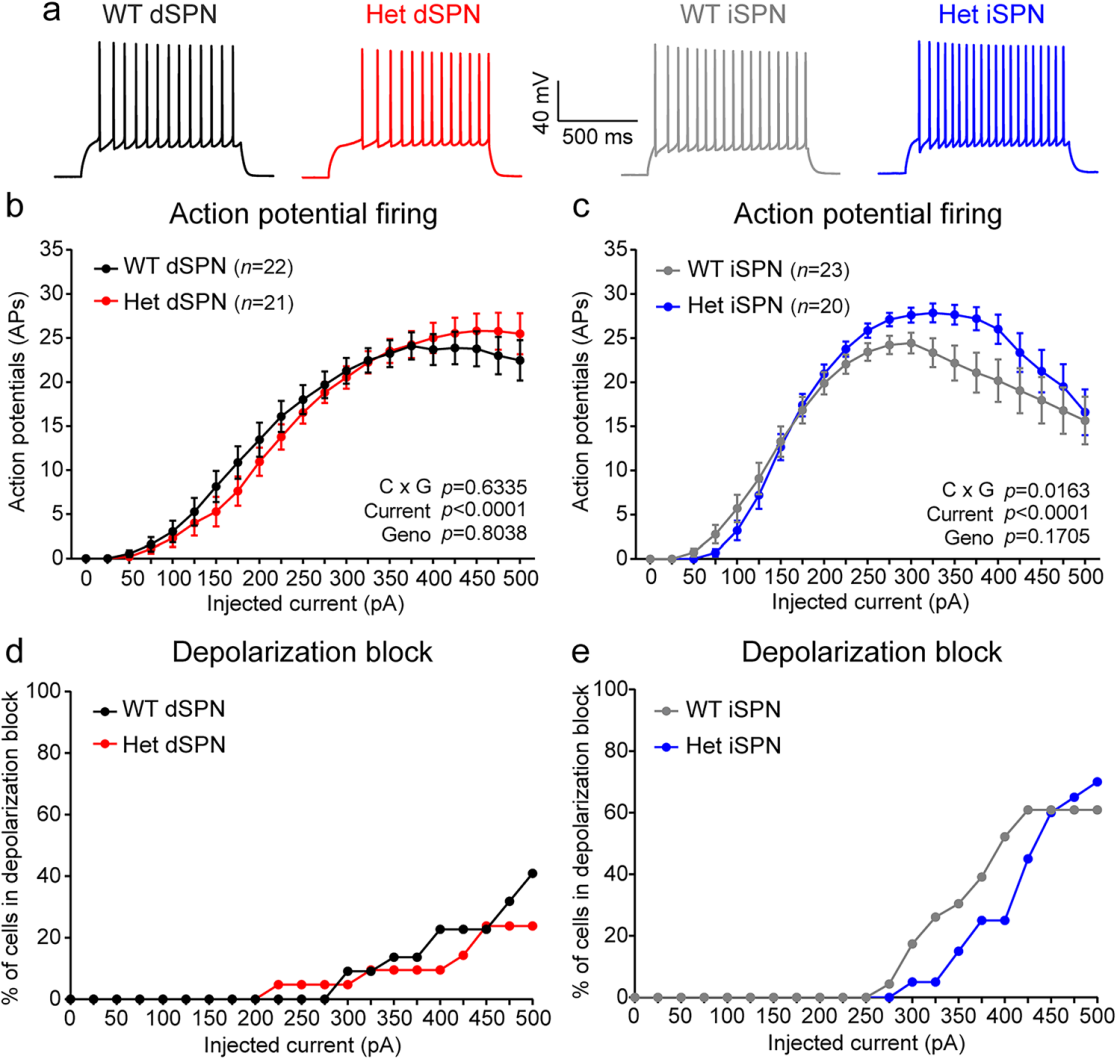
*Syngap1* haploinsufficiency alters iSPN excitability by reducing the likelihood of depolarization block. ***a***, Example action potentials elicited by a 200 pA current step in dSPNs (left) and iSPNs (right) of the indicated genotypes. ***b***, ***c***, Quantification (mean ± SEM) of action potential firing as a function of injected current for dSPNs (***b***) and iSPNs (***c***). ***b***, Input–output curve for *Syngap1* WT (*Syngap1^+/+^;D2-GFP−*, *n *= 22 neurons from 7 mice) and Het (*Syngap1^+/lx-st^*;*D2-GFP−*, *n* = 21 neurons from 6 mice) dSPNs. Two-way repeated-measures ANOVA: current × genotype *p *= 0.6335, *F*_(20,820)_ = 0.8646; current *p *< 0.0001, *F*_(1.545,63.35)_ = 110.2; genotype *p *= 0.8038, *F*_(1,41)_ = 0.0626; subject *p *< 0.0001, *F*_(41,820)_ = 7.548. ***c***, Input–output curve for *Syngap1* WT (*Syngap1^+/+^;D2-GFP+*, *n *= 23 neurons from 8 mice) and Het (*Syngap1^+/lx-st^*;*D2-GFP*+, *n* = 20 neurons from 7 mice) iSPNs. Two-way repeated-measures ANOVA: current × genotype *p *= 0.0163, *F*_(20,820)_ = 1.809; current *p *< 0.0001, *F*_(1.487,60.99)_ = 96.84; genotype *p *= 0.1705, *F*_(1,41)_ = 1.947; subject *p *< 0.0001, *F*_(41,820)_ = 8.937. ***d***, ***e***, Percentage of dSPNs (***d***) and iSPNs (***e***) of each genotype entering depolarization block at each current step. *n* is the same as for panels ***b*** (dSPNs) and ***c*** (iSPNs). See also Extended Data Table 3-1.

10.1523/JNEUROSCI.1264-23.2024.d1 Table 3-1 **Summary of individual parameters for electrophysiology experiments.** Mean +/- SEM of individual parameters for the electrophysiology experiments in Figures 3, 4, and 10. Sample sizes for each experiment are indicated in the respective figure legend. Download Table 3-1, DOCX file.

While SynGAP has been traditionally studied as a synaptic regulator, a recent study indicates that loss of *Syngap1* also impacts intrinsic excitability in developing cortical excitatory neurons through a synapse-independent mechanism (Arora et al., 2022). We therefore investigated how reduction of SynGAP affects the intrinsic membrane properties of SPNs. In current-clamp recordings, dSPNs from *Syngap1* Het mice showed no significant differences in action potential firing in response to depolarizing current steps (Fig. 3*g*,*i*) and had normal membrane resistance and capacitance (Extended Data Table 3-1). Action potential (AP) height and half-width were also not significantly altered (Extended Data Table 3-1). In contrast, iSPN-Het cells displayed altered intrinsic excitability evidenced by increased firing in response to depolarizing current, specifically at large current steps (Fig. 3*h*,*j*). Other membrane and AP properties were not significantly different in iSPN-Hets compared with WT (Extended Data Table 3-1).

To further explore these findings, we repeated the input–output experiments with finer resolution in the current steps (Fig. 4*a–c*). We observed a similar trend whereby Het dSPNs did not differ significantly in their firing response to current injection but Het iSPNs showed enhanced firing at higher current steps (Fig. 4*b*,*c*). The increased firing at steps >250 pA was likely driven by fewer iSPN-Het cells entering depolarization block compared with WT (Fig. 4*d*,*e*). There were no significant differences in rheobase, threshold, AP shape, or passive membrane properties in Het iSPNs compared with WT (Extended Data Table 3-1). These data show that loss of *Syngap1* alters the excitability of striatal neurons, with a selective effect on the firing of indirect pathway neurons.

### SynGAP reduction induces hyperactivity in the open field

**Figure 5.**
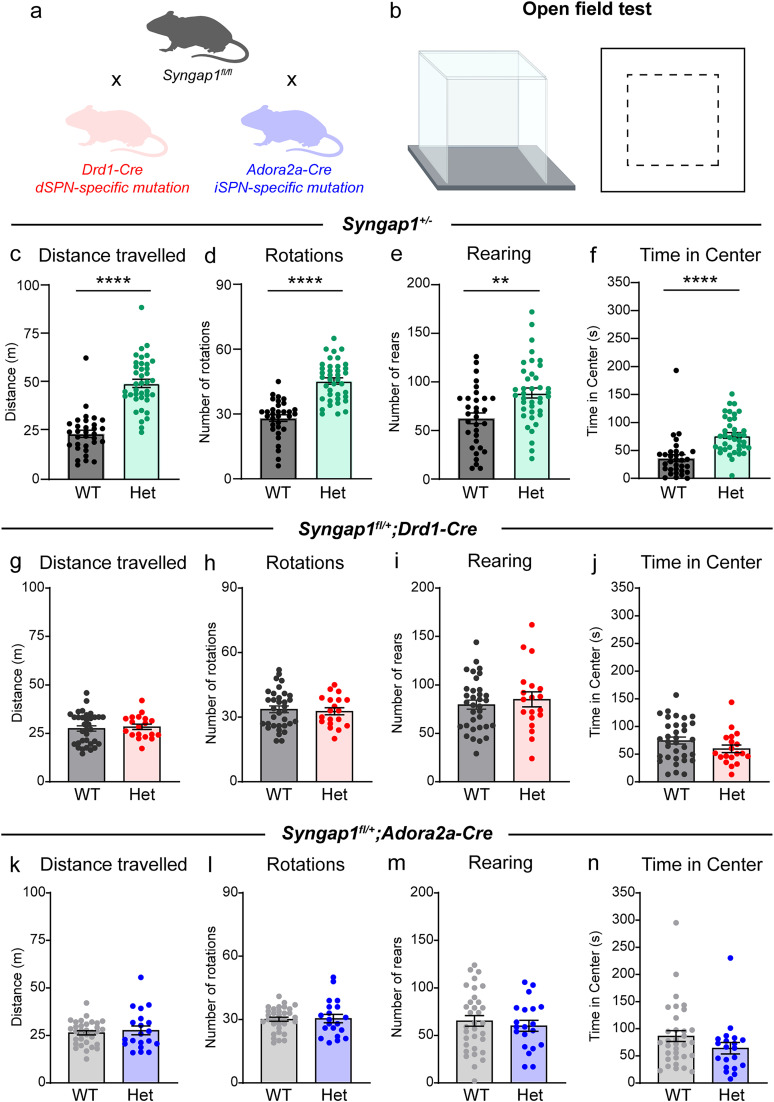
Global SynGAP reduction leads to hyperactivity in the open field. ***a***, Schematic of the Cre mouse lines used in combination with the *Syngap1^fl/fl^* line to achieve cell-type specific knock-out, created with BioRender.com. ***b***, Schematic of the open-field arena from the side view (left) and top view (right), created with BioRender.com. Dashed square represents the center of the open field. ***c–f***, Quantification of open-field parameters for *Syngap1^+/+^* (WT, *n *= 32) and *Syngap1^+/lx-st^* (Het, *n *= 39) mice. ***c***, Total distance traveled, *p *< 0.0001, *U *= 70. ***d***, Number of rotations, *p *< 0.0001, *U *= 97. ***e***, Number of rears, *p *= 0.0016, *U *= 354.5. ***f***, Time in center area, *p *< 0.0001, *U *= 171. ***g–j***, Quantification of open-field parameters for *Syngap1^+/+^;Drd1-Cre*^+^ or *Syngap1^+/+ or fl/+ or fl/fl^;Drd1-Cre^−^* (WT, *n *= 34) and *Syngap1^fl/+^;Drd1-Cre^+^* (Het, *n *= 19) mice. ***g***, Total distance traveled, *p *= 0.7758, *U *= 307. ***h***, Number of rotations, *p *= 0.8866, *U *= 315. ***i***, Number of rears, *p *= 0.6228, *U *= 296. ***j***, Time in center area, *p *= 0.1479, *U *= 244.5. ***k–n***, Quantification of open-field parameters for *Syngap1^+/+^;Adora2a-Cre*^+^ or *Syngap1^+/+ or fl/+ or fl/fl^;Adora2a-Cre^−^* (WT, *n *= 33) and *Syngap1^fl/+^;Adora2a-Cre^+^* (Het, *n *= 20) mice. ***k***, Total distance traveled, *p *= 0.9457, *U *= 326. ***l***, Number of rotations, *p *= 0.7812, *U *= 314.5. ***m***, Number of rears, *p *= 0.6328, *U *= 303.5. ***n***, Time in center area, *p *= 0.1217, *U *= 245. For panels ***c–n***, bars represent mean ± SEM and dots represent individual mice. All statistical comparisons are two-tailed Mann–Whitney tests.

*SYNGAP1*-related NDD has a variety of manifestations, including ASD, hyperexcitability, and motor abnormalities (Parker et al., 2015; Vlaskamp et al., 2019). We therefore tested whether haploinsufficiency of *Syngap1* in mice leads to similar behavioral alterations. In addition, to determine whether SynGAP loss from dSPNs or iSPNs is sufficient to drive behavioral changes, we generated conditional knock-out mice (Clement et al., 2012) in which *Syngap1* was selectively deleted from either dSPNs of the dorsal striatum (*Syngap1^fl^;Drd1-*Cre, EY217 GENSAT founder line; Gong et al., 2007) or iSPNs (*Syngap1^fl^*; *Adora2a-*Cre; Gong et al., 2007) in the dorsal and ventral striatum (Fig. 5*a*). The EY217 *Drd1*-Cre founder line was used to isolate primarily dorsal striatal neurons and avoid deletion of *Syngap1* from cortical neurons as occurs in other *Drd1*-Cre lines (Benthall et al., 2021).

We first assessed novelty-induced exploration and general locomotor activity in the open field (Fig. 5*b*). Global *Syngap1* heterozygous mice traveled a longer distance, exhibited a greater number of rotations and rears, and spent more time in the center of the arena compared with littermate WT mice (Fig. 5*c–f*). This replicates the hyperactivity commonly reported in *Syngap1* mouse models (Guo et al., 2009; Muhia et al., 2009; Nakajima et al., 2019). We repeated this experiment in *Syngap1^fl/+^;Drd1-*Cre mice and *Syngap1^fl/+^;Adora2a-*Cre mice to test whether SPNs contribute to hyperactivity. In dSPN-specific Het mice, we observed no changes in distance traveled, number of rotations, number of rears, or time spent in the center area (Fig. 5*g–j*). iSPN-specific haploinsufficiency of *Syngap1* also did not significantly affect open-field behavior (Fig. 5*k–n*). This suggests that the hyperactivity observed in global *Syngap1* Het mice cannot be reproduced by loss of *Syngap1* in dSPNs or iSPNs alone. It may be that striatum-wide disruption of *Syngap1* is necessary to induce hyperactivity. Alternatively, locomotor hyperactivity could be driven by SynGAP reduction in other cell types, such as cortical or hippocampal neurons (Clement et al., 2012; Ozkan et al., 2014).

### SynGAP reduction does not alter social approach behavior

**Figure 6.**
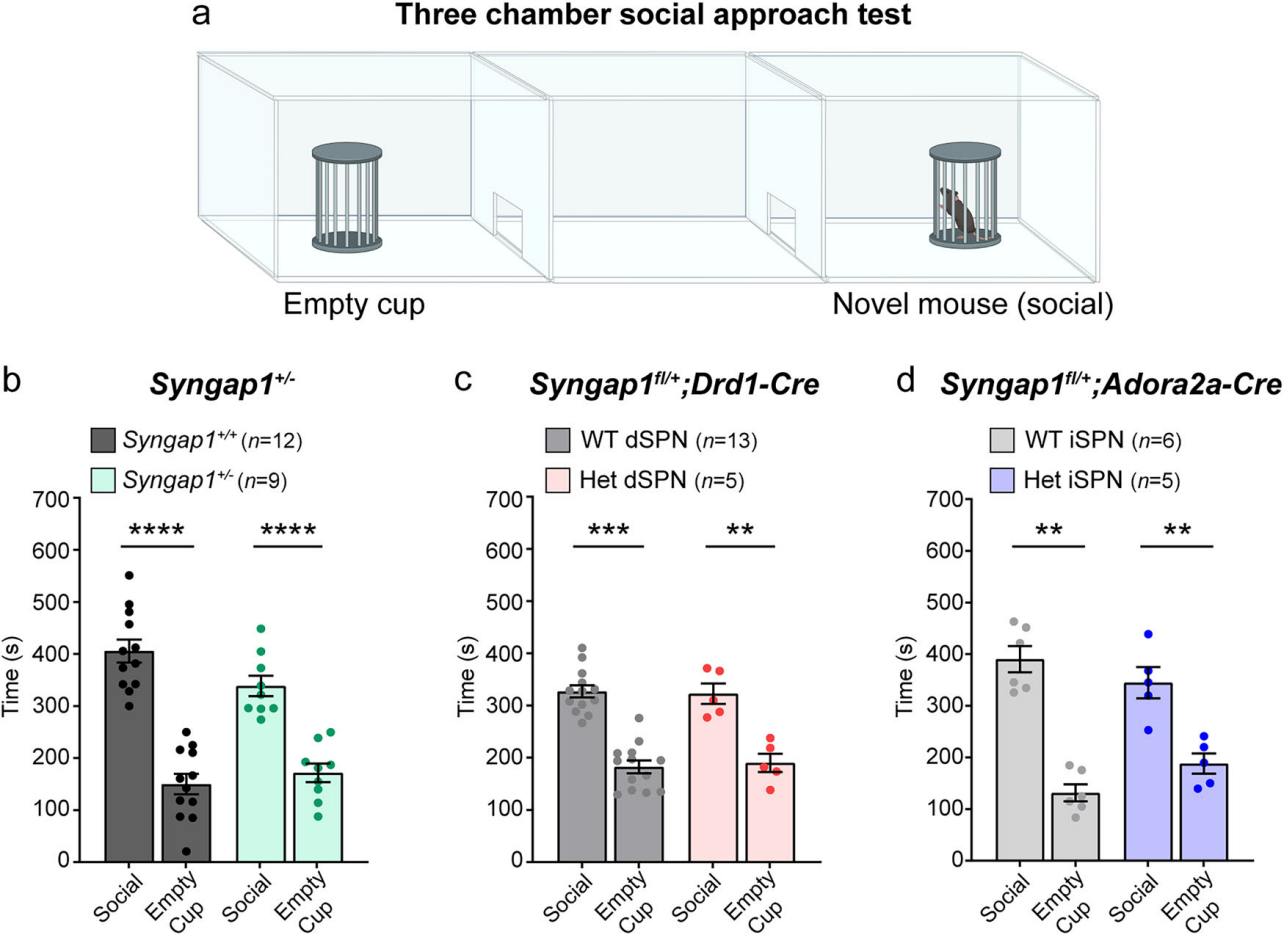
Social approach behavior is not significantly affected by reduction of SynGAP. ***a***, Schematic of the three-chamber social approach test, created with BioRender.com. ***b***, Quantification of time spent in the chamber with the novel mouse (social) or novel object (empty cup) for *Syngap1^+/+^* (WT, *n *= 12) and *Syngap1^+/lx-st^* (Het, *n *= 9) mice. Two-tailed Mann–Whitney tests: WT social versus empty, *p *< 0.0001, *U *= 0; Het social versus empty, *p *< 0.0001, *U *= 0. ***c***, Quantification of social approach for *Syngap1^+/+^;Drd1-Cre*^+^ or *Syngap1^+/+ or fl/+ or fl/fl^;Drd1-Cre^−^* (WT, *n *= 13) and *Syngap1^fl/+^;Drd1-Cre*^+^ (Het, *n *= 5) mice. Mann–Whitney tests: WT social versus empty, *p *< 0.0001, *U *= 1; Het social versus empty, *p *= 0.0079, *U *= 0. ***d***, Quantification of social approach for *Syngap1^+/+^;Adora2a-Cre*^+^ or *Syngap1^+/+ or fl/+ or fl/fl^;Adora2a-Cre^−^* (WT, *n *= 6) and *Syngap1^fl/+^;Adora2a-Cre*^+^ (Het, *n *= 5) mice. Mann–Whitney tests: WT social versus empty, *p *= 0.0022, *U *= 0; Het social versus empty, *p *= 0.0079, *U *= 0. For all panels, bars represent mean ± SEM and dots represent values for individual mice.

We next investigated phenotypes related to the core ASD domains of altered social behavior and restricted, repetitive patterns of behavior. To assay sociability, we used the three-chamber social approach test in which a test mouse can choose to spend time investigating a novel mouse or an object (Fig. 6*a*; Nadler et al., 2004). Mice with global haploinsufficiency of *Syngap1* showed normal social approach demonstrated by significantly more time spent in the chamber with the novel mouse versus an empty cup (Fig. 6*b*). Similarly, mice with dSPN- or iSPN-specific reduction of *Syngap1* also exhibited a preference for the chamber with the novel mouse (Fig. 6*c*,*d*). These results suggest that haploinsufficiency of *Syngap1* does not strongly affect social approach and are consistent with previous studies, which also found normal sociability in *Syngap1* Het mice (Guo et al., 2009; Nakajima et al., 2019). These findings in mouse models align with caregiver accounts, indicating that social functioning may be less impacted than other domains in individuals with *SYNGAP1*-related NDD (Wright et al., 2022).

### Loss of SynGAP alters motor performance in the rotarod test

A common finding across mouse models with mutations in ASD risk genes is altered motor performance on the accelerating rotarod test (Cording and Bateup, 2023). In several models, performance is enhanced and, in some cases, striatal-specific deletion of the ASD risk gene is sufficient to increase motor learning (Rothwell et al., 2014; Platt et al., 2017; Benthall et al., 2021). However, other mouse models show a deficit in rotarod performance (Wang et al., 2016; X. Yin et al., 2021), which may reflect motor impairments that are observed in a subset of individuals with ASD (Provost et al., 2007).

**Figure 7.**
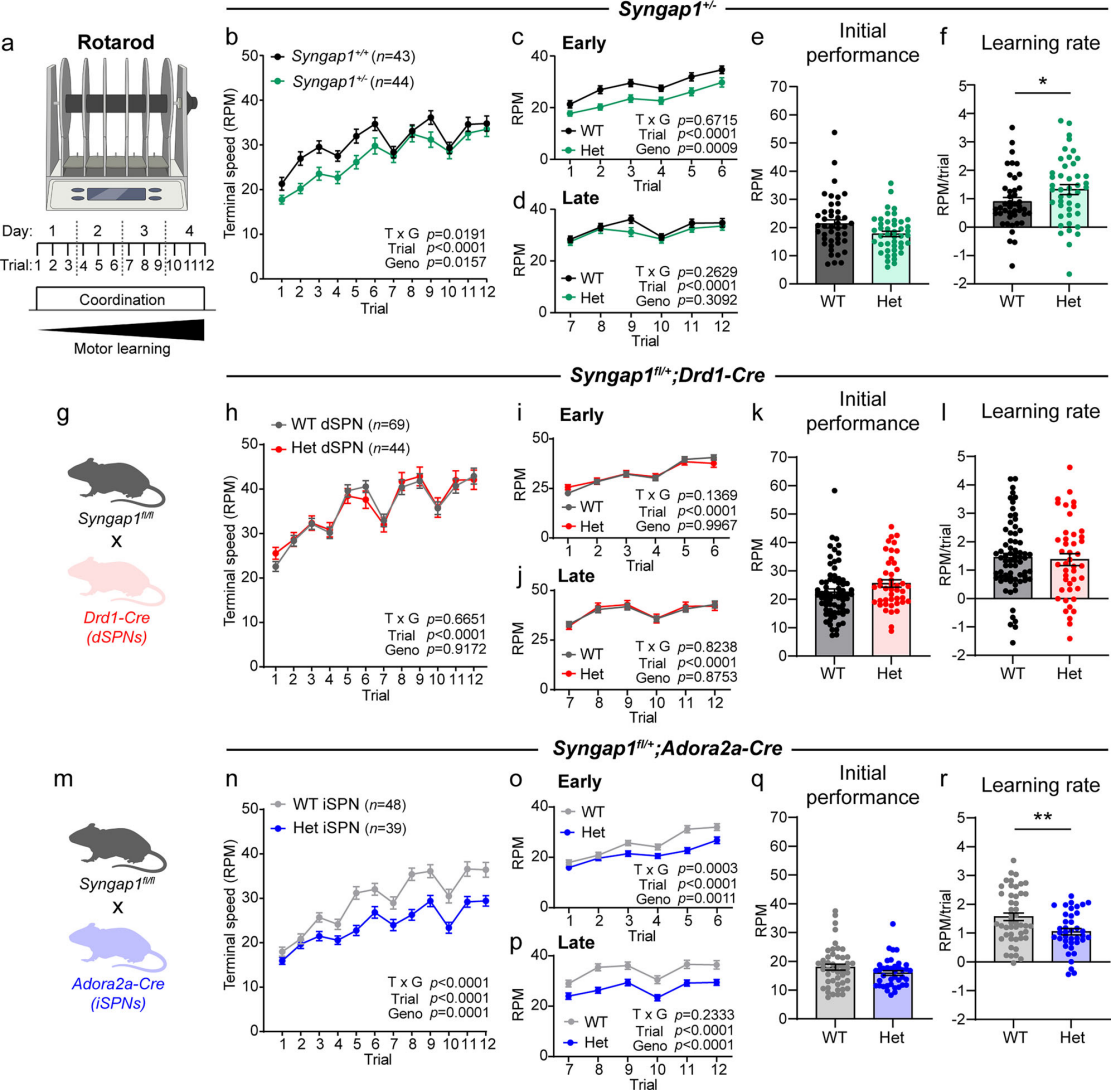
*Syngap1* haploinsufficiency alters motor behavior in the accelerating rotarod. ***a***, Schematic of the rotarod apparatus and experimental design. ***b–f***, Rotarod performance for *Syngap1^+/+^* (WT, *n *= 43) and *Syngap1^+/lx-st^* (Het, *n *= 44) mice. ***b***, Rotarod performance across all trials, measured as terminal speed (mean ± SEM). Two-way repeated-measures ANOVA: trial × genotype *p *= 0.0191, *F*_(11,935)_ = 2.084; trial *p *< 0.0001, *F*_(6.451,548.3)_ = 34.61; genotype *p *= 0.0157, *F*_(1,85)_ = 6.073; subject *p *< 0.0001, *F*_(85,935)_ = 10.00. ***c***, Performance in Trials 1–6. Two-way repeated-measures ANOVA: trial × genotype *p *= 0.6715, *F*_(5,425)_ = 0.6371; trial *p *< 0.0001, *F*_(4.042,343.6)_ = 40.43; genotype *p *= 0.0009, *F*_(1,85)_ = 11.83; subject *p *< 0.0001, *F*_(85,425)_ = 7.450. ***d***, Performance in Trials 7–12. Two-way repeated-measures ANOVA: trial × genotype *p *= 0.2629, *F*_(5,425)_ = 1.300; trial *p *< 0.0001, *F*_(4.360,370.6)_ = 14.48; genotype *p *= 0.3092, *F*_(1,85)_ = 1.047; subject *p *< 0.0001, *F*_(85,425)_ = 8.900. ***e***, Performance on Trial 1. Two-tailed Mann–Whitney test, *p *= 0.0630, *U *= 727. ***f***, Learning rate, measured as the slope of performance from Trial 1 to Trial 12 for each mouse. Mann–Whitney test, *p *= 0.0436, *U *= 708.5. ***g***, Schematic of the mouse cross to generate dSPN conditional Het mice. ***h–l***, Rotarod performance for *Syngap1^+/+^;Drd1-Cre*^+^ or *Syngap1^+/+ or fl/+ or fl/fl^;Drd1- Cre^−^* (WT, *n *= 69) and *Syngap1^fl/+^;Drd1-Cre^+^* (Het, *n *= 44) mice. ***h***, Rotarod performance across all trials (mean ± SEM). Two-way repeated-measures ANOVA: trial × genotype *p *= 0.6651, *F*_(11,1,221)_ = 0.7754; trial *p *< 0.0001, *F*_(6.878,763.4)_ = 50.72; genotype *p *= 0.9172, *F*_(1,111)_ = 0.0109; subject *p *< 0.0001, *F*_(111,1,221)_ = 11.04. ***i***, Performance in Trials 1–6. Two-way repeated-measures ANOVA: trial × genotype *p *= 0.1369, *F*_(5,555)_ = 1.682; trial *p *< 0.0001, *F*_(4.097,454.8)_ = 61.21; genotype *p *= 0.9967, *F*_(1,111)_ < 0.0001; subject *p *< 0.0001, *F*_(111,555)_ = 5.875. ***j***, Performance in trials 7–12. Two-way repeated-measures ANOVA: trial × genotype *p *= 0.8238, *F*_(5,555)_ = 0.4355; trial *p *< 0.0001, *F*_(4.730,525.1)_ = 28.15; genotype *p *= 0.8753, *F*_(1,111)_ = 0.0247; subject *p *< 0.0001, *F*_(111,555)_ = 11.28. ***k***, Performance on trial 1. Mann–Whitney test, *p *= 0.0731, *U *= 1,214. ***l***, Learning rate. Mann–Whitney test, *p *= 0.7393, *U *= 1,461. ***m***, Schematic of the mouse cross to generate iSPN conditional Het mice. ***n–r***, Rotarod performance for *Syngap1^+/+^;Adora2a-Cre*^+^ or *Syngap1^+/+ or fl/+ or fl/fl^;Adora2a-Cre^−^* (WT, *n *= 48) and *Syngap1^fl/+^;Adora2a-Cre^+^* (Het, *n *= 39) mice. ***n***, Rotarod performance across all trials, (mean ± SEM). Two-way repeated-measures ANOVA: trial × genotype *p *< 0.0001, *F*_(11,935)_ = 3.938; trial *p *< 0.0001, *F*_(6.984,593.7)_ = 70.03; genotype *p *< 0.0001, *F*_(1,85)_ = 16.38; subject *p *< 0.0001, *F*_(85,935)_ = 14.53. ***o***, Performance in Trials 1–6. Two-way repeated-measures ANOVA: trial × genotype *p *= 0.0003, *F*_(5,425)_ = 4.712; trial *p *< 0.0001, *F*_(3.822,324.9)_ = 57.58; genotype *p *= 0.0011, *F*_(1,85)_ = 11.49; subject *p *< 0.0001, *F*_(85,455)_ = 6.278. ***p***, Performance in Trials 7–12. Two-way repeated-measures ANOVA: trial × genotype *p *= 0.2333, *F*_(5,425)_ = 1.373; trial *p *< 0.0001, *F*_(4.298,365.4)_ = 28.12; genotype *p *< 0.0001, *F*_(1,85)_ = 16.82; subject *p *< 0.0001, *F*_(85,425)_ = 13.65. ***q***, Performance on trial 1. Mann–Whitney test, *p *= 0.1790, *U *= 778. ***r***, Learning rate. Mann–Whitney test, *p *= 0.0087, *U *= 630.5. For panels ***e***, ***f***, ***k***, ***l***, ***q***, and ***r***, bars represent mean ± SEM and dots represent values for individual mice. Panels ***a***, ***g***, and ***m*** were created with BioRender.com.

To examine how *Syngap1* haploinsufficiency impacts motor coordination and striatal-dependent motor learning, we tested mice on the accelerating rotarod (5–80 rpm) with three trials per day across 4 consecutive testing days (Fig. 7*a*). Global *Syngap1* Het mice exhibited overall decreased performance measured across all 12 trials (Fig. 7*b*), with a trend toward decreased initial performance on trial one (Fig. 7*e*). These results replicate a prior report of decreased rotarod performance in an independent *Syngap1^+/−^* mouse model (Nakajima et al., 2019). Notably, when we analyzed the first two and last two testing days separately, we found that Het mice had a significant performance deficit in the early trials (#1–6), which recovered in later trials (7–12; Fig. 7*c*,*d*). This resulted in a small but significant increase in motor learning rate, measured as the slope of performance for each mouse from Trial 1 to Trial 12 (Fig. 7*f*). Notably, this profile is similar to that previously reported in *16p11.2^+/−^* mice, which harbor an ASD-associated copy number variant (X. Yin et al., 2021).

We tested dSPN-specific *Syngap1* Het mice in the same task and found no significant differences in initial performance, early versus late performance, or motor learning compared with WT littermates (Fig. 7*g–l*). In contrast, mice with iSPN-specific reduction of *Syngap1* had overall reduced performance, in line with the global heterozygous mice (Fig. 7*m*,*n*). Like global heterozygotes, iSPN-Het mice showed reduced performance in early trials (Fig. 7*o*), but performance on trial one was not significantly altered (Fig. 7*q*). Unlike the global heterozygotes, however, the performance deficit in early trials persisted into the later trials (Fig. 7*p*) and iSPN-Het mice had, on average, a reduced learning rate (Fig. 7*r*). Together, these results show that motor performance on the accelerating rotarod is impaired in mice with global *Syngap1* haploinsufficiency and that the striatal indirect pathway may be a contributor to this phenotype.

### SynGAP reduction affects goal-directed behavior

**Figure 8.**
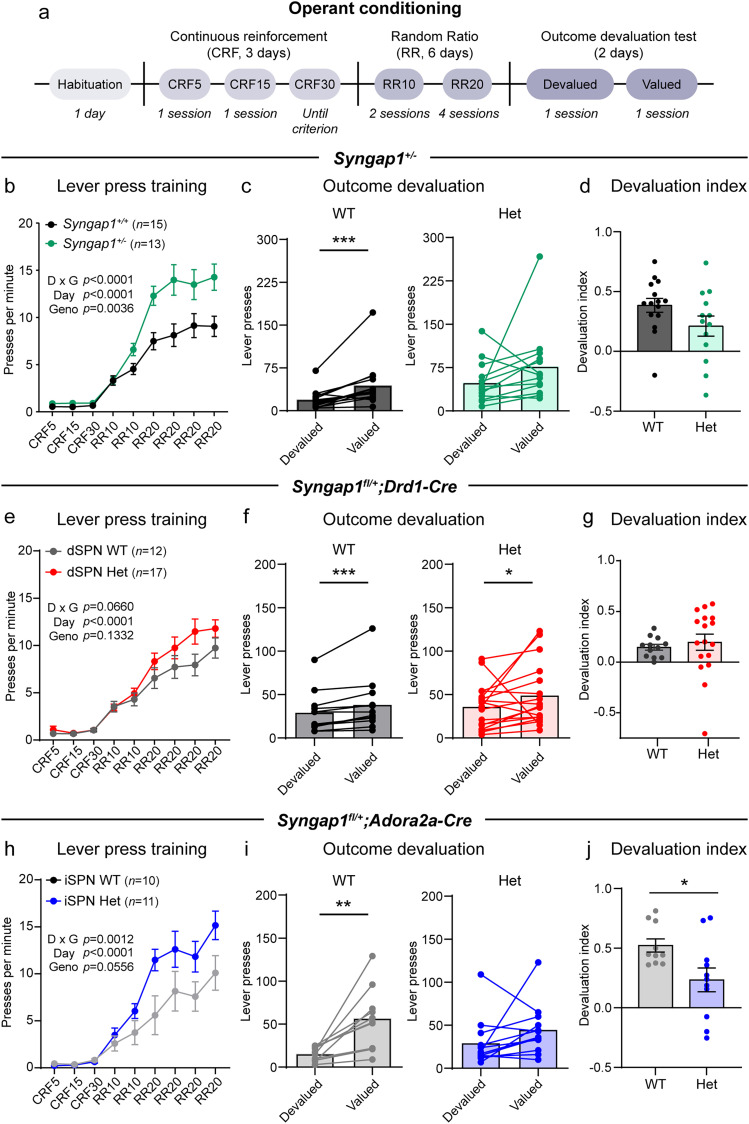
SynGAP reduction leads to increased lever pressing and altered goal-directed responding in an operant conditioning task. ***a***, Schematic and timeline of the random ratio operant conditioning task. ***b–d***, Operant task performance for *Syngap1^+/+^* (WT, *n *= 15) and *Syngap1^+/lx-st^* (Het, *n *= 13) mice. ***b***, Number of lever presses per minute across sessions (mean ± SEM). CRF, continuous reinforcement schedule; RR, random ratio schedule. Two-way repeated-measures ANOVA: day × genotype *p *< 0.0001, *F*_(8,208)_ = 5.814; day *p *< 0.0001, *F*_(1.896,49.30)_ = 92.69; genotype *p *= 0.0036, *F*_(1,26)_ = 10.24; subject *p *< 0.0001, *F*_(26,208)_ = 5.680. ***c***, Number of lever presses on the devalued versus valued session for each mouse. Bars represent the mean. Wilcoxon matched-pairs signed rank test: WT devalued versus valued, *p *= 0.0002; Het devalued versus valued, *p *= 0.0942. ***d***, Devaluation index (mean ± SEM, dots represent values for individual mice). Two-tailed Mann–Whitney test, *p *= 0.0796, *U *= 59. ***e–g***, Operant task performance for *Syngap1^+/+^;Drd1-Cre*^+^ or *Syngap1^+/+ or fl/+ or fl/fl^;Drd1-Cre^−^* (WT, *n *= 12) and *Syngap1^fl/+^;Drd1-Cre^+^* (Het, *n *= 17) mice. ***e***, Number of lever presses per minute across sessions (mean ± SEM). Two-way repeated-measures ANOVA: day × genotype *p *= 0.0660, *F*_(8,216)_ = 1.870; day *p *< 0.0001, *F*_(2.065,55.76)_ = 76.77; genotype *p *= 0.1332, *F*_(1,27)_ = 2.397; subject *p *< 0.0001, *F*_(27,216)_ = 6.007. ***f***, Number of lever presses on the devalued versus valued session for each mouse. Bars represent the mean. Wilcoxon matched-pairs signed rank test: WT devalued versus valued, *p *= 0.0010; Het devalued versus valued, *p *= 0.0241. ***g***, Devaluation index (mean ± SEM, dots represent values for individual mice). Mann–Whitney test, *p *= 0.2405, *U *= 75. ***h–j***, Operant task performance for *Syngap1^+/+^;Adora2a-Cre*^+^ or *Syngap1^+/+ or fl/+ or fl/fl^;Adora2a-Cre^−^* (WT, *n *= 10) and *Syngap1^fl/+^;Adora2a-Cre^+^* (Het, *n *= 11) mice. ***h***, Number of lever presses per minute across sessions (mean ± SEM). Two-way repeated-measures ANOVA: day × genotype *p *= 0.0012, *F*_(8,152)_ = 3.432; day *p *< 0.0001, *F*_(2.304,43.77)_ = 51.94; genotype *p *= 0.0556, *F*_(1,19)_ = 4.157; subject *p *< 0.0001, *F*_(19,152)_ = 7.531. ***i***, Number of lever presses on the devalued versus valued session for each mouse. Bars represent the mean. Wilcoxon matched-pairs signed rank test: WT devalued versus valued, *p *= 0.0020; Het devalued versus valued, *p *= 0.0723. ***j***, Devaluation index (mean ± SEM, dots represent values for individual mice). Mann–Whitney test, *p *= 0.0101, *U *= 19.

**Figure 9.**
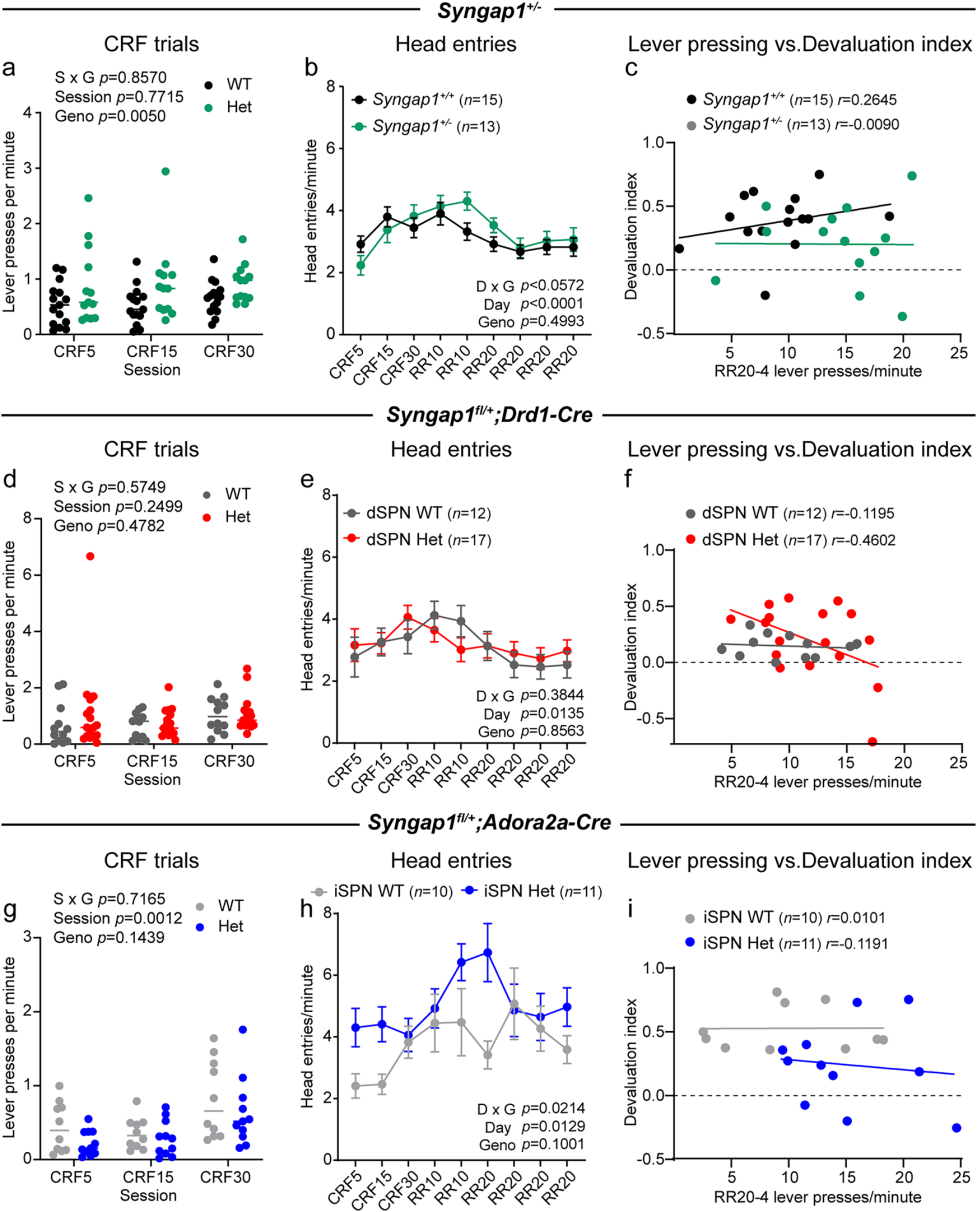
Additional analysis of operant conditioning behavior. ***a–c***, Operant task performance for *Syngap1^+/+^* (WT, *n *= 15) and *Syngap1^+/lx-st^* (Het, *n *= 13) mice. ***a***, Lever presses per minute for the three continuous reinforcement (CRF) trials (CRF5, 15 and 30). Dots represent values for individual mice and horizontal lines represent the group median. Two-way repeated-measures ANOVA *p* values are shown: genotype × session, *p *= 0.8570, *F*_(2,52)_ = 0.1548; session, *p *= 0.7715, *F*_(1.856,48.25)_ = 0.2396; genotype, *p *= 0.0050, *F*_(1,26)_ = 9.416; subject, *p *= 0.3727, *F*_(26,52)_ = 1.103. ***b***, Rate of head entries across training sessions (mean ± SEM). Mixed-effects analysis: day × genotype, *p *= 0.0572, *F*_(8,205)_ = 1.930; day, *p *< 0.0001, *F*_(5.179,132,7)_ = 8.125; genotype, *p *= 0.4993, *F*_(1,26)_ = 0.4695. ***c***, Correlation between lever press rate on the fourth day of RR20 training and the devaluation index for each mouse. WT Pearson's *r *= 0.2645, *p *= 0.3408; Het Pearson's *r *= −0.0090, *p *= 0.9767. ***d–f***, Operant task performance for *Syngap1^+/+^;Drd1-Cre*^+^ or *Syngap1^+/+ or fl/+ or fl/fl^;Drd1-Cre^−^* (WT, *n *= 12) and *Syngap1^fl/+^;Drd1- Cre^+^* (Het, *n *= 17) mice. ***d***, Lever presses per minute for the CRF5, 15 and 30 trials. Two-way repeated-measures ANOVA *p* values are shown: genotype × session, *p *= 0.5749, *F*_(2,54)_ = 0.5593; session, *p *= 0.2499, *F*_(1.253,33.82)_ = 1.413; genotype, *p *= 0.4782, *F*_(1,27)_ = 0.5172; subject, *p *= 0.0434, *F*_(27,54)_ = 1.730. ***e***, Rate of head entries across training sessions (mean ± SEM). Repeated-measures two-way ANOVA: day × genotype, *p *= 0.3844, *F*_(8,216)_ = 1.071; day, *p *= 0.0135, *F*_(3.204,86.50)_ = 3.668; genotype, *p *= 0.8563, *F*_(1,27)_ = 0.0335; subject, *p *< 0.0001, *F*_(27,216)_ = 6.727. ***f***, Correlation between lever press rate on the fourth day of RR20 training and the devaluation index for each mouse. dSPN WT Pearson's *r *= −0.1195, *p *= 0.7115; dSPN Het Pearson's *r *= −0.4602, *p *= 0.0630. ***g–i***, Operant task performance for *Syngap1^+/+^;Adora2a-Cre*^+^ or *Syngap1^+/+ or fl/+ or fl/fl^;Adora2a-Cre^−^* (WT, *n *= 10) and *Syngap1^fl/+^;Adora2a-Cre^+^* (Het, *n *= 11) mice. ***g***, Lever presses per minute for the CRF5, 15 and 30 trials. Two-way repeated-measures ANOVA *p* values are shown: genotype × session, *p *= 0.7165, *F*_(2,38)_ = 0.3363; session, *p *= 0.0012, *F*_(1.298,24.67)_ = 11.34; genotype, *p *= 0.1439, *F*_(1,19)_ = 2.323; subject, *p *= 0.0757, *F*_(19,38)_ = 1.723. ***h***, Rate of head entries across training sessions (mean ± SEM). Repeated-measures two-way ANOVA: day × genotype, *p *= 0.0214, *F*_(8,152)_ = 2.338; day, *p *= 0.0129, *F*_(3.125,59.37)_ = 3.844; genotype, *p *= 0.1001, *F*_(1,19)_ = 2.989; subject, *p *< 0.0001, *F*_(19,152)_ = 8.802. ***i***, Correlation between lever press rate on the fourth day of RR20 training and the devaluation index for each mouse. iSPN WT Pearson's *r *= 0.0101, *p *= 0.9979; iSPN Het Pearson's *r *= −0.1192, *p *= 0.7271.

ASD is characterized by behavioral inflexibility. To determine whether this may reflect the formation of motor habits, which are maintained even when the outcome of the action is no longer rewarded, we assessed the behavior of *Syngap1* mouse models in a random ratio (RR) operant conditioning task (Figs. 8, 9; Hilario et al., 2007; Renteria et al., 2021). In this task, mice are food-restricted and initially trained through a series of continuous reinforcement trials (CRF) where for every lever press, the mouse is rewarded with a food pellet. Following this initial training, mice switch to a RR reinforcement schedule where each lever press has a 1 in 10 (RR10) or 1 in 20 (RR20) chance of reward presentation. In the outcome devaluation test, mice are given *ad libitum* access to the food reward resulting in the outcome (food) of the action (lever press) becoming devalued. The mice then enter an unrewarded 5 min probe trial during which lever pressing is quantified. In the valued probe trial session, mice are given *ad libitum* access to a different reward (sucrose water), which should control for satiety but not devalue the food reinforcer. The devaluation index ([lever presses valued state − lever presses devalued state] / [lever presses valued state + lever presses devalued state]) is computed as a metric of goal-directed behavior with higher scores signifying greater sensitivity to outcome devaluation. Mice that have adopted a more flexible, goal-oriented strategy are expected to have an index closer to one, while mice who have developed a devaluation-insensitive motor habit will have an index closer to zero.

We tested global *Syngap1* Het mice in the RR task (Fig. 8*a*) and plotted lever presses per minute across sessions. *Syngap1* Het mice pressed the lever significantly more compared with their WT littermates (Fig. 8*b*), indicating strong acquisition of lever pressing behavior in this task. The increased lever pressing was already apparent during the CRF trials (Fig. 9*a*), but there was no difference between genotypes in the rate of head entries into the reward magazine during training (Fig. 9*b*). In outcome devaluation testing, WT mice pressed the lever significantly more in the valued probe trial compared with the devalued probe trial (Fig. 8*c*), demonstrating goal-directed behavior, as expected. However, *Syngap1* Het mice exhibited more erratic lever pressing across the two probe trials (Fig. 8*c*), with some mice exhibiting lack of sensitivity to devaluation. As a result, the average devaluation index was lower for *Syngap1* Het mice compared with WT (Fig. 8*d*), although this did not reach statistical significance. The rate of lever pressing on the last day of training (fourth session of RR20) was not significantly correlated with the devaluation index for each mouse (Fig. 9*c*), suggesting that the lack of consistent sensitivity to devaluation in *Syngap1* Het mice was not due to their increased lever pressing. Overall, this suggests a shift in goal-directed behavior toward more habitual responding in *Syngap1* heterozygous mice.

We next tested conditional *Syngap1* Het mice in the RR test. Consistent with the rotarod and open-field tests, we observed no significant differences in lever pressing across sessions in dSPN-specific *Syngap1* Het mice compared with controls (Figs. 8*e*, 9*d*), although we noted a slight trend toward increased pressing in later trials in dSPN-Het mice. Both dSPN-WT and dSPN-Het mice displayed increased lever pressing on the valued day compared with the devalued day, demonstrating sensitivity to outcome devaluation (Fig. 8*f*,*g*). There was no difference in head entries or correlation between lever pressing and devaluation index for the dSPN mice (Fig. 9*e*,*f*). In contrast, mice with iSPN-specific haploinsufficiency of *Syngap1* exhibited increased lever pressing during training, similar to global heterozygotes (Fig. 8*h*). This effect was not present in the CRF trials (Fig. 9*g*) but became apparent in the RR sessions. There was also a significant interaction between head entries and training session in the iSPN-Hets (Fig. 9*h*). Plotting lever presses across the devalued and valued probe trials revealed similar inconsistent pressing in iSPN-Hets as seen for the global heterozygotes, as well as a lower average devaluation index (Figs. 8*i*,*j*, 9*i*). Together, these results show that global SynGAP reduction leads to altered goal-directed behavior, which can be reproduced by reduction of *Syngap1* in iSPNs.

### Genetic rescue of SynGAP in indirect pathway cells normalizes spine and behavioral phenotypes

**Figure 10.**
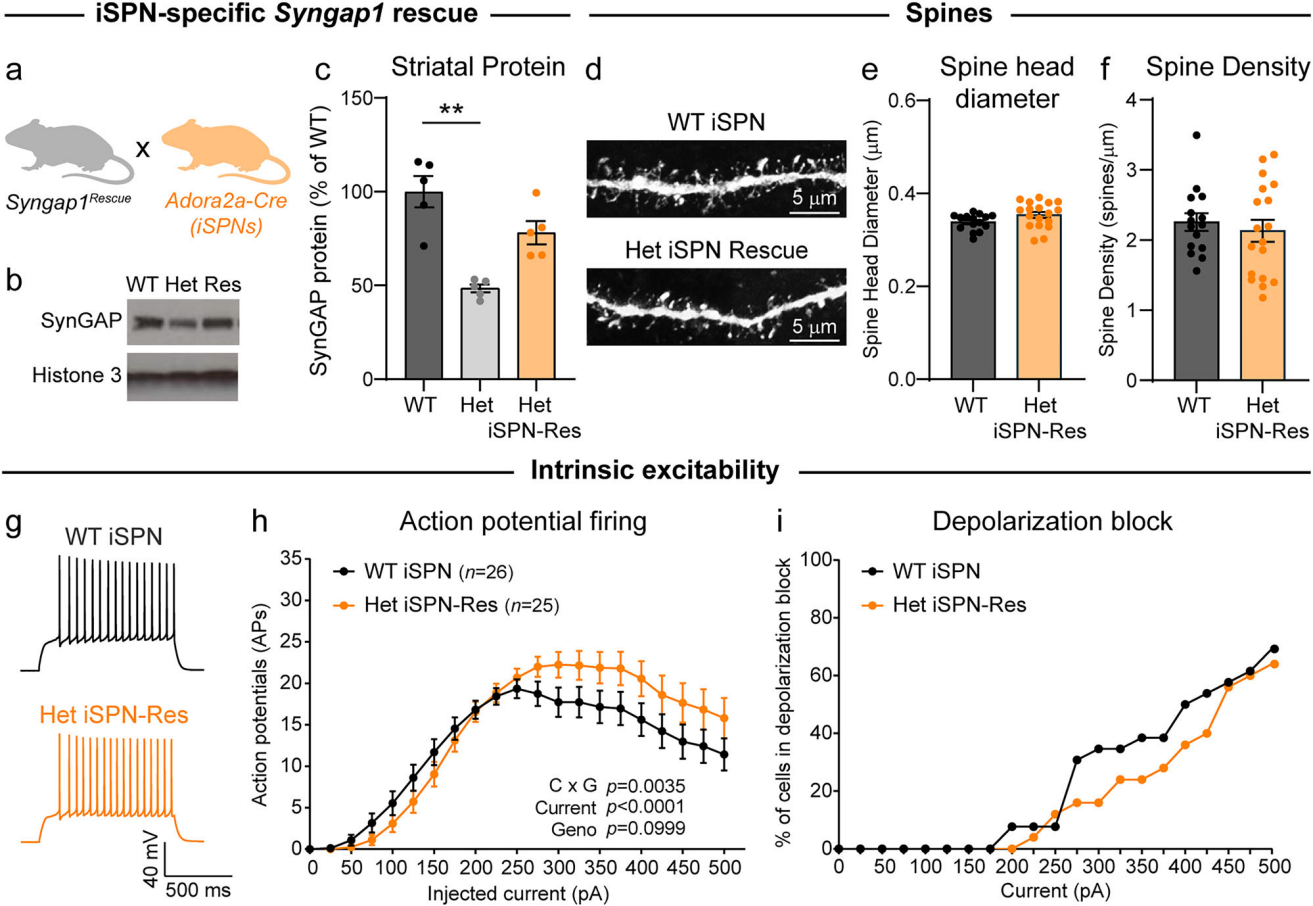
iSPN-specific genetic rescue of *Syngap1* prevents spine phenotypes. ***a***, Schematic of the mouse cross used to generate iSPN-specific rescue of *Syngap1*, created with BioRender.com. ***b***, Representative Western blots (WB) of striatal lysates from *Syngap1^+/+^;Adora2a-Cre^+^* (WT), *Syngap1^+/lx-st^;Adora2a-Cre^−^* (Het) and *Syngap1^+/lx-st^;Adora2a-Cre^+^* (Het iSPN-Res, “Res”) mice showing SynGAP protein and Histone 3 loading control. This experiment was repeated five times. ***c***, Quantification of striatal SynGAP protein levels from *Syngap1* WT, Het, and Het iSPN rescue mice, expressed as a percent of WT (mean ± SEM, dots represent values from individual mice, *n = *5 mice per genotype). Kruskal–Wallis test, *p *= 0.0002, K–W statistic = 10.82; Dunn's multiple-comparisons tests: WT versus Het, *p *= 0.0034; WT versus Res, *p *= 0.6880; Het versus Res, *p *= 0.1209. ***d***, Representative images of dendritic spines from a WT iSPN and Het iSPN rescue neuron. Scale bars, 5 µm. ***e***, Quantification of spine head diameter in *Syngap1* WT iSPNs (*n *= 15 neurons from 4 mice) and Het rescue iSPNs (*n *= 18 neurons from 4 mice) (mean ± SEM, dots represent values for individual neurons). Two-tailed unpaired *t* test, *p *= 0.0699, *t *= 1.877, df = 31. ***f***, Quantification of spine density in *Syngap1* WT iSPNs and Het rescue iSPNs (mean ± SEM, dots represent values for individual neurons, *n* is the same as for panel ***e***). Two-tailed unpaired *t* test, *p *= 0.5523, *t *= 0.6009, df = 31. ***g***, Example traces of action potentials elicited by a 200 pA current step in *Syngap1^+/+^;Adora2a-Cre^+^*;Ai9 (WT) and *Syngap1^+/lx-st^;Adora2a-Cre^+^*;Ai9 (Het iSPN-Res) iSPNs. ***h***, Quantification (mean ± SEM) of action potentials elicited by different current steps for *Syngap1* WT (*n *= 26 neurons from 5 mice) and Het iSPN-Res (*n* = 25 neurons from 4 mice) iSPNs. Two-way repeated-measures ANOVA: current × genotype *p *= 0.0035, *F*_(20,980)_ = 2.083; current *p *< 0.0001, *F*_(1.610,78.91)_ = 51.75; genotype *p *= 0.0999, *F*_(1,49)_ = 2.812; subject *p *< 0.0001, *F*_(49,980)_ = 4.517. ***i***, Percentage of iSPNs of each genotype entering depolarization block at each current step. *n* is the same as for panel ***h***. See also Extended Data Table 3-1.

Our results suggest that iSPNs are affected by *Syngap1* haploinsufficiency and contribute to behavioral phenotypes. Therefore, we asked whether it was possible to prevent cellular and behavioral phenotypes in *Syngap1* Het mice by genetic restoration of *Syngap1* in iSPNs only. To do this, we bred *Syngap1^+/lx-st^* mice to *Adora2a-*Cre mice to reinstate *Syngap1* expression in iSPNs beginning in embryonic development (Fig. 10*a*). We confirmed that this strategy led to ∼75% of WT expression of SynGAP protein in the striatum, as expected given that dSPNs and iSPNs each make up ∼45–50% of the total neuron population (Fig. 10*b*,*c*). We assessed whether iSPN spine number and morphology were normalized in *Syngap1^+/lx-st^*;*Adora2a*-Cre^+^ (Het iSPN-rescue) mice. We found no significant differences in spine density or spine head diameter, rescuing the previously observed changes in global *Syngap1* Het mice (Fig. 10*d–f*). We also tested whether altered intrinsic excitability could be rescued by expression of *Syngap1* in iSPNs. Interestingly, while we observed a slight amelioration of the phenotype, Het iSPN-rescue neurons still exhibited increased firing at large current steps compared with WT SPNs (Fig. 10*g–i*). This suggests that the effects of *Syngap1* loss on iSPN excitability may, in part, be mediated by noncell autonomous circuit-level changes, given that the rest of the cells in the brain remain haploinsufficient for *Syngap1* in this model.

**Figure 11.**
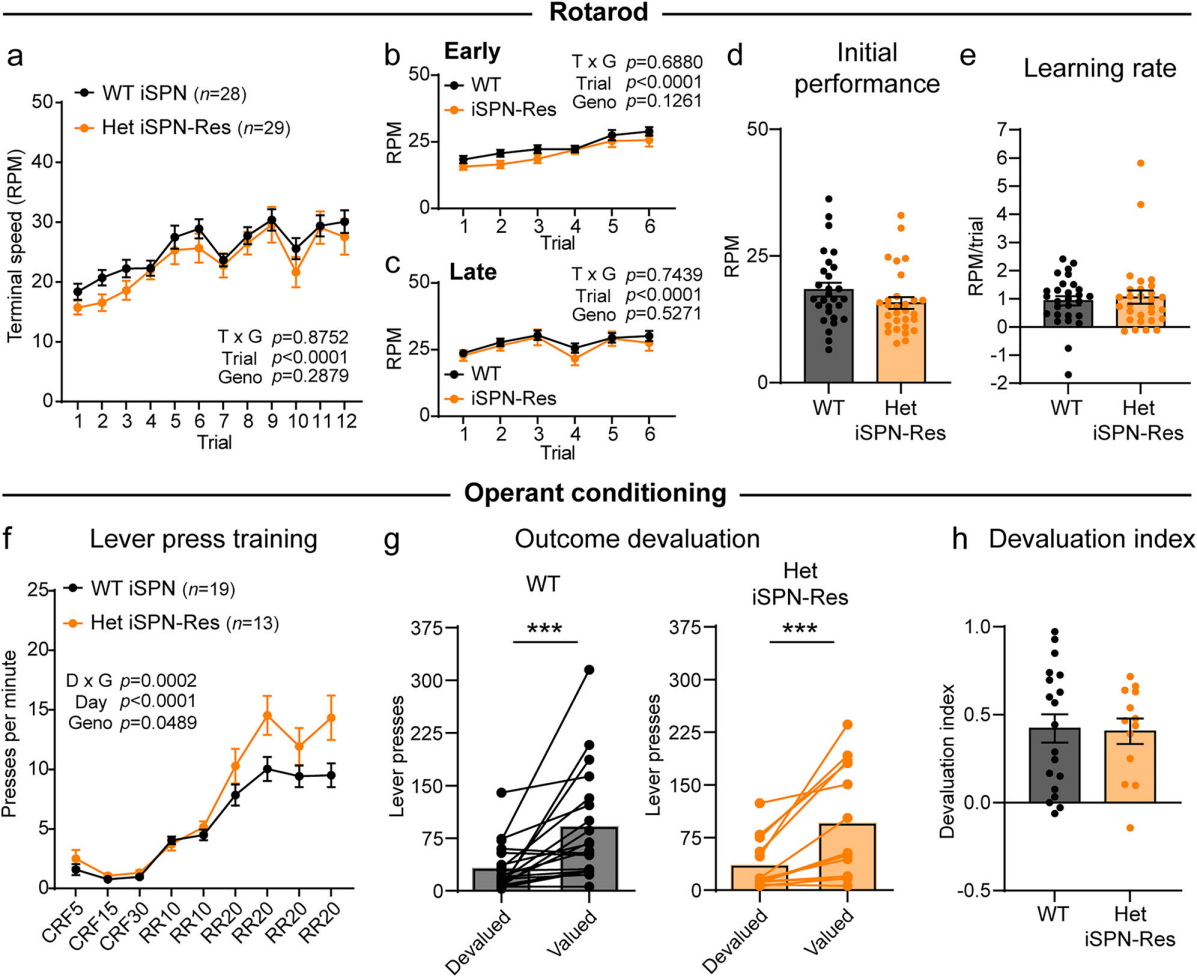
Genetic rescue of SynGAP in indirect pathway cells ameliorates behavioral phenotypes. ***a–e***, Rotarod performance for *Syngap1^+/+^;Adora2a-Cre^+^* (WT, *n *= 28) and *Syngap1^+/lx-st^;Adora2a-Cre^+^* (Het iSPN-Res, mice, *n *= 29). ***a***, Rotarod performance across all trials, measured as terminal speed (mean ± SEM). Two-way repeated-measures ANOVA: trial × genotype *p *= 0.8752, *F*_(11,605)_ = 0.5416; trial *p *< 0.0001, *F*_(5.967,328.2)_ = 19.91; genotype *p *= 0.2879, *F*_(1,55)_ = 1.151; subject *p *< 0.0001, *F*_(55,605)_ = 13.06. ***b***, Performance in Trials 1–6. Two-way repeated-measures ANOVA: trial × genotype *p *= 0.6880, *F*_(5,275)_ = 0.6156; trial *p *< 0.0001, *F*_(3.371,185.4)_ = 22.38; genotype *p *= 0.1261, *F*_(1,55)_ = 2.413; subject *p *< 0.0001, *F*_(55,275)_ = 5.902. ***c***, Performance in Trials 7–12. Two-way repeated-measures ANOVA: trial × genotype *p *= 0.7439, *F*_(5,275)_ = 0.5426; trial *p *< 0.0001, *F*_(3.846,211.5)_ = 10.05; genotype *p *= 0.5271, *F*_(1,55)_ = 0.4051; subject *p *< 0.0001, *F*_(55,275)_ = 11.39. ***d***, Performance on Trial 1. Two-tailed Mann–Whitney test, *p *= 0.0755, *U *= 294.5. ***e***, Learning rate, measured as the slope of performance from Trial 1 to Trial 12 for each mouse. Mann–Whitney test, *p *= 0.5182, *U *= 365. ***f–h***, Operant task performance for *Syngap1^+/+^;Adora2a-Cre^+^* (WT, *n *= 19) and *Syngap1^+/lx-st^;Adora2a-Cre^+^* (Het iSPN-Res, mice, *n *= 13). ***f***, Number of lever presses per minute across sessions (mean ± SEM). CRF, continuous reinforcement schedule; RR, random ratio schedule. Two-way repeated-measures ANOVA: day × genotype *p *= 0.0002, *F*_(8,240)_ = 3.919; day *p *< 0.0001, *F*_(2.199,65.97)_ = 97.38; genotype *p *= 0.0489, *F*_(1,30)_ = 4.215; subject *p *< 0.0001, *F*_(30,240)_ = 7.642. ***g***, Number of lever presses on the devalued versus valued session for each mouse. Bars represent the mean. Wilcoxon matched-pairs signed rank test: WT devalued versus valued, *p *= 0.0001; Het iSPN-Res devalued versus valued, *p *= 0.0005. ***h***, Devaluation index (mean ± SEM, dots represent values for individual mice). Mann–Whitney test, *p *= 0.8798, *U *= 119.

To determine whether genetic restoration of *Syngap1* in iSPNs could prevent behavioral deficits, we tested Het iSPN-rescue mice on the accelerating rotarod and compared their performance to littermate *Syngap1^wt/wt^*;*Adora2a*-Cre^+ or −^ (WT) mice. We found no differences between WT and Het iSPN-rescue mice in overall performance or performance in the early or late trials (Fig. 11*a–c*). There were also no significant differences in initial motor coordination or learning rate (Fig. 11*d*,*e*), suggesting that iSPNs are an important player in the rotarod phenotypes observed in global Het mice (Fig. 7). Finally, we tested whether genetic rescue of *Syngap1* in iSPNs restored goal-directed behavior in the RR task. While we did observe mildly increased lever pressing in the Het iSPN-rescue mice compared with WT (Fig. 11*f*), this difference was less pronounced than what was observed in the global Het mice (Fig. 8), suggesting a partial rescue of this phenotype. iSPN-specific restoration of *Syngap1* expression also improved the consistency of goal-directed behavior, resulting in significantly higher lever pressing on the valued day compared with the devalued day and a similar devaluation index to WT mice (Fig. 11*g*,*h*). Together, these results indicate that genetic restoration of *Syngap1* in iSPNs is sufficient to prevent changes in dendritic spine morphology, motor behavior, and goal-directed responding in mice with global *Syngap1* haploinsufficiency.

## Discussion

*SYNGAP1*-related nonsyndromic intellectual disability typically results from de novo heterozygous mutations (Hamdan et al., 2009) with ∼50% of *SYNGAP1* patients exhibiting ASD symptoms (Vlaskamp et al., 2019). In addition, *SYNGAP1* has been identified as a high-confidence ASD risk gene in multiple studies (De Rubeis et al., 2014; Satterstrom et al., 2020). Although striatal circuits are strongly implicated across mouse models of ASD (Fuccillo, 2016; Li and Pozzo-Miller, 2020), the role of *SYNGAP1* in the striatum has not yet been defined. Here we show in mice that *Syngap1* is expressed in both dSPNs and iSPNs, which comprise the major output pathways of the striatum (Fig. 1). We demonstrate that SynGAP reduction affects synapse number, spine morphology, and intrinsic excitability primarily in iSPNs (Figs. 2–4). In addition, we show that *Syngap1^+/−^* mice exhibit several behavioral changes in domains that are altered in individuals with *SYNGAP1*-related NDD, including motor function and goal-directed behavior (Figs. 7–9). Notably, several of these behavioral changes can be recapitulated in mice with selective mutation of *Syngap1* in iSPNs only. These experiments identify the consequences of *Syngap1* haploinsufficiency on striatal-dependent behaviors and implicate the indirect pathway as a potential contributor to *SYNGAP1-*related NDD.

### SynGAP is expressed in striatal neurons and regulates synapses and intrinsic excitability

Previous research has primarily focused on SynGAP's function in the cortex and hippocampus (Clement et al., 2012; Ozkan et al., 2014; Aceti et al., 2015; Berryer et al., 2016). Studies assessing brain-wide SynGAP expression reported SynGAP protein in the striatum (Porter et al., 2005; Araki et al., 2020; Gou et al., 2020), but little is known about its function in striatal cell types. We found robust *Syngap1* mRNA expression in the majority (>90%) of dSPNs and iSPNs, which was present at similar levels in both cell types (Fig. 1). SynGAP is localized to the postsynaptic density, and several studies have reported alterations in dendritic spine number or morphology resulting from *Syngap1* mutations (Vazquez et al., 2004; Clement et al., 2012; Aceti et al., 2015; Arora et al., 2022). Consistent with these results, we found that *Syngap1* haploinsufficiency led to decreased spine density and increased spine head diameter in SPNs (Fig. 2). iSPNs were predominantly affected with dSPNs exhibiting no or only subtle changes, though the mechanism for this cell type-specific effect remains unknown. We note that several other studies examining the consequences of ASD-related mutations in the striatum have reported cell type-specific effects (Rothwell et al., 2014; Benthall et al., 2018, 2021; Davatolhagh and Fuccillo, 2021; Cording et al., 2024). iSPNs and dSPNs are molecularly and developmentally distinct (Heiman et al., 2008; Tinterri et al., 2018), which may account for their different responses to disease-associated insults. It is also possible that neuromodulators such as dopamine exert differential effects on synaptic function in dSPNs versus iSPNs due to their distinct expression of dopamine receptors (Tritsch and Sabatini, 2012).

To functionally assess synapses, we recorded mEPSCs and found that, consistent with the spine results, Het iSPNs had a decrease in mEPSC frequency (Fig. 3). Given that we observed reduced spine number, this suggests fewer glutamatergic inputs to these cells. Surprisingly, mEPSC amplitude was also decreased in Het iSPNs, which was unexpected given the increased spine head size (Fig. 3). SynGAP is thought to restrain AMPAR-mediated transmission; therefore, SynGAP reduction is expected to increase synaptic strength (Rumbaugh et al., 2006; McMahon et al., 2012). Since our recordings were done in adults, it is possible that homeostatic changes occurred over development, which led to a decoupling of spine size and synaptic strength.

A recent report showed that loss of *Syngap1* affects not only spines and synapses, but also intrinsic membrane properties (Arora et al., 2022). We investigated this and found an iSPN-specific alteration in intrinsic excitability, with increased firing in response to larger depolarizing currents (Figs. 3, 4). With increasing current injection, SPNs are known to enter a phase of reduced action potential firing, termed “depolarization block.” This likely occurs due to the inactivation of voltage-gated sodium channels. Our results suggest that *Syngap1* haploinsufficiency may reduce the inactivation probability of voltage-gated sodium channels at higher current injections in iSPNs. dSPNs and iSPNs have different intrinsic membrane properties and excitability (Gertler et al., 2008; Planert et al., 2013), which may account for why loss of *Syngap1* affected the excitability of one cell type but not the other.

### SynGAP reduction alters striatal-dependent behaviors

*SYNGAP1*-related NDD presents with a host of symptoms including hyperexcitability, motor abnormalities, ASD, and other behavioral manifestations (Parker et al., 2015; Kilinc et al., 2018; Vlaskamp et al., 2019). Previous studies using *Syngap1*^+/−^ mice found that SynGAP reduction leads to hyperactivity in the open field and a reduction in avoidance behavior, indicated by an increase in time spent in the center region (Guo et al., 2009; Muhia et al., 2009). We replicated both findings in our experiments but showed that they are likely driven either by the coordinated activity of both SPN subtypes or by regions outside of the striatum, as dSPN or iSPN-specific reduction of *Syngap1* was not sufficient to induce these changes (Fig. 5). In support of this, selective reduction of *Syngap1* in excitatory forebrain neurons can induce open-field hyperactivity, whereas loss of *Syngap1* in Gad2-expressing inhibitory neurons (which includes SPNs) does not (Ozkan et al., 2014).

Alterations in motor learning have been reported across multiple mouse models of ASD (Cording and Bateup, 2023), with some models showing enhanced motor learning and others exhibiting deficits, which may reflect the motor impairments observed in some individuals with ASD (Chukoskie et al., 2013). We found that *Syngap1^+/−^* mice had reduced rotarod performance, which was most pronounced in the earlier trials (Fig. 7). Notably, performance in later trials was similar to WT mice. This suggests that Het mice had an initial motor impairment that could be overcome with repeated training on the task. In conditional heterozygous mice, loss of *Syngap1* from iSPNs only was sufficient to impair performance on the rotarod, manifesting as reduced performance in both early and late trials. Individuals with *SYNGAP1* mutations have motor alterations including unstable gait, low muscle tone, poor coordination, and altered fine motor skills (Vlaskamp et al., 2019; Wright et al., 2022), which could be related to the rotarod phenotypes observed in mice.

Habitual actions that persist independently of outcome value are mediated by the DLS, while the dorsomedial striatum (DMS) is important for goal-directed actions, which are guided by outcome and action–reward contingencies (H. H. Yin et al., 2004, 2005; Balleine and O'Doherty, 2010). In disorders associated with repetitive, inflexible behaviors, such as ASD, there may be a shift in action strategy toward more habitual responding (Boyd et al., 2012; Uddin, 2021; Tian et al., 2022). We therefore assessed how SynGAP reduction affects outcome devaluation in a self-paced operant conditioning task (Hilario et al., 2007; Renteria et al., 2021). We found that both global *Syngap1^+/−^* and iSPN-specific Het mice exhibited increased lever pressing during RR training (Fig. 8). This suggests that *Syngap1* Hets readily learn and acquire reward-related lever pressing behavior. Increased lever pressing could either reflect rapid motor habit formation or an increased motivation to pursue rewarded actions. A previous study reported increased “vigor” in learned operant lever pressing behavior in *Syngap1^+/−^* mice, with no change in motivation as assessed by a progressive ratio test (Muhia et al., 2009). Interestingly, this study also reported impaired extinction following progressive ratio testing in *Syngap1^+/−^* mice, which indicates persistent responding in the absence of reward. Taken together with our results showing that iSPN-specific Hets do not have increased locomotory activity, this suggests that enhanced lever pressing is likely due to increased learning and not simply hyperactivity.

In outcome devaluation testing, global heterozygotes and iSPN-Het mice did not consistently show sensitivity to devaluation, as reflected by the large variability in the devaluation index across mice (Fig. 8). We note that dSPN-Het mice also had a low devaluation index; however, this was not significantly different from the WT mice in this cohort. While global and iSPN-specific *Syngap1* disruption altered goal-directed responding, the nature of this effect is more complex than a straightforward shift from one action strategy to another, as some mice showed sensitivity to devaluation while others did not and some even pressed the lever more on the devalued day. Taken together, the overall reduced devaluation index in *Syngap1^+/−^* mice suggests a disruption to flexible, goal-directed behavior, which may reflect the behavioral inflexibility experienced by individuals with *SYNGAP1*-related NDD.

In terms of linking the cell physiology phenotypes, we observed with the behavioral phenotypes, further experiments will be needed to determine how the combination of reduced excitatory synapse number and strength together with increased excitability translates into activation of iSPNs during behavior. In particular, the mEPSCs we recorded represent an aggregate of glutamatergic synapses. In the dorsal striatum, excitatory inputs come primarily from the cortex and thalamus, but also the amygdala (Pan et al., 2010). Since cortical synapses are thought to be made onto the spine head, while thalamic synapses are preferentially on the dendritic shaft (Dube et al., 1988), it is possible that one set of inputs could be strengthened while the other could be weakened. In addition, striatal interneurons provide powerful context-specific control over SPN firing (Fino et al., 2018) and whether or how inhibitory synapses are affected by *Syngap1* haploinsufficiency remains to be determined. Further investigation using in vivo recording approaches will be required to determine how complex changes in cellular and synaptic physiology resulting from *Syngap1* haploinsufficiency combine to impact the activity of the direct and indirect pathways during learning.

### Prevention of phenotypes with genetic rescue of *Syngap1* in iSPNs

The results summarized above demonstrate a collection of iSPN-driven phenotypes resulting from *Syngap1* haploinsufficiency. As cell type-specific heterozygous mice do not recapitulate the disease state in which all cells are affected, we sought to test to what extent phenotypes could be prevented by genetic restoration of *Syngap1* in iSPNs only. We used a mouse model in which Cre recombinase causes removal of a stop cassette and re-expression of the WT *Syngap1* gene in a cell type-specific manner (Clement et al., 2012). Changes in iSPN spine density and spine head diameter were prevented in the iSPN rescue mice demonstrating that these are cell autonomous changes resulting from SynGAP loss in iSPNs (Fig. 10). Interestingly, alterations in intrinsic excitability persisted to some extent in Het iSPN rescue mice, indicating a potential non-cell autonomous mechanism for this change (Fig. 10). In terms of behavior, motor performance and goal-directed responding were normalized in the iSPN-specific rescue mice (Fig. 11). This suggests that genetic restoration of *Syngap1* in iSPNs alone is sufficient to prevent some phenotypes of *Syngap1* haploinsufficiency and confirms the indirect pathway as an important mediator of SynGAP-related behavioral phenotypes.

### Limitations and future directions

The findings described here provide a starting point to further explore the synaptic and behavioral changes induced by *Syngap1* reduction in the striatum. Importantly, an open question is why iSPNs are more strongly impacted than dSPNs, when *Syngap1* is expressed in both. Here we focused on the DLS since changes in striatal activity in this region are associated with the formation of motor habits (Gremel and Costa, 2013), which may underlie restricted and repetitive behaviors in ASD (Fuccillo, 2016). Since we did observe changes in goal-directed behavior, future studies could examine additional striatal regions and circuits including the DMS.

In terms of technical aspects, we note that *Drd1-Cre* line used here (GENSAT EY217 founder line) is not fully penetrant, with more expression in the dorsal versus ventral striatum (Benthall et al., 2021). This is not the case for the *Adora2a-Cre* line which shows equivalent expression across all striatal sub-regions (Benthall et al., 2021). The EY217 founder line was chosen over other *Drd1-Cre* lines as it exhibits more selective striatal expression, with minimal expression in the cortex, which would confound our results. The behaviors explored in this study are known to be mediated by the dorsal striatum (H. H. Yin et al., 2004, 2005, 2009), and we have previously shown that the EY217 mouse line targets enough dSPNs to induce cell type-specific changes in striatal-dependent motor behaviors (Benthall et al., 2021).

Finally, we note that this study was not sufficiently powered to explore sex differences, although it has been reported that SynGAP may regulate synaptic properties in a sex-specific manner (Mastro et al., 2020). Sex differences have not been reported in human patient populations thus far (Vlaskamp et al., 2019), and our preliminary analyses do not point toward major sex differences in the experiments performed.
