# Supplementary material for: Haploinsufficiency of Syngap1 in Striatal Indirect Pathway Neurons Alters Motor and Goal-Directed Behaviors in Mice
Source: J Neurosci. 2024 Oct 2;44(48):e1264232024. doi: 10.1523/JNEUROSCI.1264-23.2024 (PMC11604145; doi:10.1523/JNEUROSCI.1264-23.2024)
Supplement: Table 3-1 — Summary of individual parameters for electrophysiology experiments. Mean +/- SEM of individual parameters for the electrophysiology experiments in Figures 3, 4, and 10. Sample sizes for each experiment are indicated in the respective figure legend. Download Table 3-1, DOCX file. [file jneuro-44-e1264232024-s001.docx]

**Extended Data**

**Haploinsufficiency of *Syngap1* in striatal indirect pathway neurons alters motor and goal-directed behaviors in mice**

*Abbreviated title: Syngap1 modulates striatal physiology and behavior*

Laura M. Haetzel^1,*^, Jillian Iafrati^2,*^, Katherine R. Cording^1^, Mahmoud Farhan^2^, Sasan D. Noveir^2^, Gavin Rumbaugh^3,4^, Helen S. Bateup^1,2,5,6^

Number of Extended Data Tables: 1

**Extended Data Table 3-1. Summary of individual parameters for electrophysiology experiments.**

Mean +/- SEM of individual parameters for the electrophysiology experiments in Figures 3, 4, and 10. Sample sizes for each experiment are indicated in the respective figure legend.

| **Fig. 3 mEPSCs** | | | | |
| --- | --- | --- | --- | --- |
| **Parameter** | **Genotype** | **Cell type** | **Mean** | **SEM** |
| Avg. amplitude (pA) | WT | dSPN | 11.61 | 0.363 |
|  | Het |  | 11.22 | 0.402 |
|  | WT | iSPN | 11.83 | 0.196 |
|  | Het |  | 10.71 | 0.233 |
| Avg. frequency (Hz) | WT | dSPN | 2.536 | 0.424 |
|  | Het |  | 3.570 | 0.503 |
|  | WT | iSPN | 2.653 | 0.223 |
|  | Het |  | 1.932 | 0.311 |
| Series resistance (MOhm) | WT | dSPN | 10.58 | 0.343 |
|  | Het |  | 10.44 | 0.521 |
|  | WT | iSPN | 9.940 | 0.268 |
|  | Het |  | 9.941 | 0.436 |
| Membrane resistance (MOhm) | WT | dSPN | 341.9 | 27.39 |
|  | Het |  | 388.1 | 34.12 |
|  | WT | iSPN | 431.9 | 27.97 |
|  | Het |  | 618.0 | 55.05 |
| Capacitance (pF) | WT | dSPN | 143.3 | 10.04 |
|  | Het |  | 142.4 | 7.834 |
|  | WT | iSPN | 139.8 | 4.458 |
|  | Het |  | 104.7 | 6.892 |
| **Fig 3. intrinsic excitability** | | | | |
| **Parameter** | **Genotype** | **Cell type** | **Mean** | **SEM** |
| Rheobase current (pA) | WT | dSPN | 177.5 | 14.62 |
|  | Het |  | 152.1 | 12.45 |
|  | WT | iSPN | 91.11 | 8.852 |
|  | Het |  | 83.33 | 5.601 |
| Threshold (mV) | WT | dSPN | -40.61 | 1.568 |
|  | Het |  | -43.36 | 1.182 |
|  | WT | iSPN | -45.58 | 0.923 |
|  | Het |  | -46.85 | 1.044 |
| Action potential height (mV) | WT | dSPN | 93.36 | 1.620 |
|  | Het |  | 94.90 | 2.345 |
|  | WT | iSPN | 92.87 | 1.248 |
|  | Het |  | 95.83 | 1.152 |
| Action potential half-width (ms) | WT | dSPN | 1.943 | 0.039 |
|  | Het |  | 2.029 | 0.050 |
|  | WT | iSPN | 1.993 | 0.041 |
|  | Het |  | 2.074 | 0.038 |
| After hyperpolarization (mV) | WT | dSPN | -87.34 | 0.478 |
|  | Het |  | -79.65 | 2.256 |
|  | WT | iSPN | -83.18 | 1.236 |
|  | Het |  | -79.88 | 0.930 |
| Resting membrane potential (mV) | WT | dSPN | -86.12 | 0.866 |
|  | Het |  | -80.85 | 1.814 |
|  | WT | iSPN | -82.49 | 1.076 |
|  | Het |  | -78.42 | 0.962 |
| Membrane resistance (MOhm) | WT | dSPN | 177.4 | 14.70 |
|  | Het |  | 134.4 | 9.991 |
|  | WT | iSPN | 292.1 | 22.72 |
|  | Het |  | 228.3 | 24.52 |
| Capacitance (pF) | WT | dSPN | 122.1 | 5.667 |
|  | Het |  | 115.7 | 10.66 |
|  | WT | iSPN | 103.7 | 5.410 |
|  | Het |  | 93.96 | 5.410 |
| **Fig. 4 intrinsic excitability (smaller current steps)** | | | | |
| **Parameter** | **Genotype** | **Cell type** | **Mean** | **SEM** |
| Rheobase current (pA) | WT | dSPN | 140.9 | 12.90 |
|  | Het |  | 151.2 | 11.76 |
|  | WT | iSPN | 107.6 | 7.769 |
|  | Het |  | 116.3 | 5.524 |
| Threshold (mV) | WT | dSPN | -44.44 | 0.576 |
|  | Het |  | -44.68 | 0.636 |
|  | WT | iSPN | -44.30 | 1.039 |
|  | Het |  | -45.16 | 0.616 |
| Action potential height (mV) | WT | dSPN | 103.8 | 0.961 |
|  | Het |  | 102.6 | 1.548 |
|  | WT | iSPN | 102.1 | 0.963 |
|  | Het |  | 102.3 | 1.021 |
| Action potential half-width (ms) | WT | dSPN | 1.979 | 0.034 |
|  | Het |  | 2.038 | 0.041 |
|  | WT | iSPN | 1.967 | 0.029 |
|  | Het |  | 1.990 | 0.031 |
| After hyperpolarization (mV) | WT | dSPN | -81.47 | 0.958 |
|  | Het |  | -81.59 | 1.056 |
|  | WT | iSPN | -81.55 | 0.785 |
|  | Het |  | -81.44 | 0.991 |
| Resting membrane potential (mV) | WT | dSPN | -81.82 | 0.941 |
|  | Het |  | -81.67 | 1.070 |
|  | WT | iSPN | -81.34 | 0.799 |
|  | Het |  | -81.86 | 0.968 |
| Membrane resistance (MOhm) | WT | dSPN | 176.4 | 20.19 |
|  | Het |  | 167.1 | 16.27 |
|  | WT | iSPN | 217.2 | 16.84 |
|  | Het |  | 195.7 | 11.21 |
| Capacitance (pF) | WT | dSPN | 116.8 | 6.607 |
|  | Het |  | 124.7 | 4.744 |
|  | WT | iSPN | 105.3 | 4.828 |
|  | Het |  | 113.3 | 3.698 |
| **Fig. 10 iSPN rescue intrinsic excitability** | | | | |
| **Parameter** | **Genotype** | **Cell type** | **Mean** | **SEM** |
| Rheobase current (pA) | WT | iSPN | 110.6 | 8.224 |
|  | Het Res iSPN |  | 127.0 | 8.155 |
| Threshold (mV) | WT |  | -42.59 | 0.725 |
|  | Het Res iSPN |  | -41.47 | 0.634 |
| Action potential height (mV) | WT |  | 98.72 | 1.092 |
|  | Het Res iSPN |  | 99.03 | 0.697 |
| Action potential half-width (ms) | WT |  | 1.932 | 0.025 |
|  | Het Res iSPN |  | 1.915 | 0.023 |
| After hyperpolarization (mV) | WT |  | -80.40 | 0.959 |
|  | Het Res iSPN |  | -80.34 | 0.933 |
| Resting membrane potential (mV) | WT |  | -80.86 | 0.952 |
|  | Het Res iSPN |  | -80.66 | 0.913 |
| Membrane resistance (MOhm) | WT |  | 243.7 | 27.74 |
|  | Het Res iSPN |  | 203.5 | 13.49 |
| Capacitance (pF) | WT |  | 102.8 | 5.186 |
|  | Het Res iSPN |  | 111.1 | 4.524 |
